# The Identification of Zebrafish Mutants Showing Alterations in Senescence-Associated Biomarkers

**DOI:** 10.1371/journal.pgen.1000152

**Published:** 2008-08-15

**Authors:** Shuji Kishi, Peter E. Bayliss, Junzo Uchiyama, Eriko Koshimizu, Jie Qi, Purushothama Nanjappa, Shintaro Imamura, Asiful Islam, Donna Neuberg, Adam Amsterdam, Thomas M. Roberts

**Affiliations:** 1 Schepens Eye Research Institute, Harvard Medical School, Boston, Massachusetts, United States of America; 2 Department of Ophthalmology, Harvard Medical School, Boston, Massachusetts, United States of America; 3 Department of Cancer Biology, Dana-Farber Cancer Institute, Boston, Massachusetts, United States of America; 4 Department of Pathology, Harvard Medical School, Boston, Massachusetts, United States of America; 5 Tokyo University of Marine Science and Technology, Tokyo, Japan; 6 College of Marine Life Science, Ocean University of China, Qingdao, China; 7 Department of Biostatistics and Computational Biology, Dana-Farber Cancer Institute, Boston, Massachusetts, United States of America; 8 Department of Biostatistics, Harvard School of Public Health, Boston, Massachusetts, United States of America; 9 Koch Institute for Integrative Cancer Research, Massachusetts Institute of Technology, Cambridge, Massachusetts, United States of America; University of Pennsylvania School of Medicine, United States of America

## Abstract

There is an interesting overlap of function in a wide range of organisms between genes that modulate the stress responses and those that regulate aging phenotypes and, in some cases, lifespan. We have therefore screened mutagenized zebrafish embryos for the altered expression of a stress biomarker, senescence-associated β-galactosidase (SA-β-gal) in our current study. We validated the use of embryonic SA-β-gal production as a screening tool by analyzing a collection of retrovirus-insertional mutants. From a pool of 306 such mutants, we identified 11 candidates that showed higher embryonic SA-β-gal activity, two of which were selected for further study. One of these mutants is null for a homologue of *Drosophila spinster*, a gene known to regulate lifespan in flies, whereas the other harbors a mutation in a homologue of the human *telomeric repeat binding factor 2* (*terf2*) gene, which plays roles in telomere protection and telomere-length regulation. Although the homozygous *spinster* and *terf2* mutants are embryonic lethal, heterozygous adult fish are viable and show an accelerated appearance of aging symptoms including lipofuscin accumulation, which is another biomarker, and shorter lifespan. We next used the same SA-β-gal assay to screen chemically mutagenized zebrafish, each of which was heterozygous for lesions in multiple genes, under the sensitizing conditions of oxidative stress. We obtained eight additional mutants from this screen that, when bred to homozygosity, showed enhanced SA-β-gal activity even in the absence of stress, and further displayed embryonic neural and muscular degenerative phenotypes. Adult fish that are heterozygous for these mutations also showed the premature expression of aging biomarkers and the accelerated onset of aging phenotypes. Our current strategy of mutant screening for a senescence-associated biomarker in zebrafish embryos may thus prove to be a useful new tool for the genetic dissection of vertebrate stress response and senescence mechanisms.

## Author Summary

By performing genetic mutant screens using senescence-associated biomarkers, we show that the zebrafish is a tractable model system for the study of aging. In vertebrate organisms, it has not previously been possible to carry out systematic screens for genes that are important for stress responses and aging in an unbiased way. However, such vertebrate models are of considerable importance, given the provocative evidence of common biochemical and functional pathways modulating stress responses and lifespan as well as aging in a wide range of organisms. Our present study has successfully employed a colorimetric high-throughput method using a senescence-associated β-galactosidase-based assay to screen for mutations that alter the stress responses in zebrafish embryos, in the hope that these might represent potential aging mutants. Subsequently, the mutations identified by embryonic senescence have indeed displayed adult aging-related phenotypes in zebrafish. Hence, our method for the identification of mutant zebrafish has the immediate potential to accelerate the discovery of novel genes and new functions relevant for our understanding of aging processes in vertebrates. Such knowledge will be essential for the ultimate development of pharmacological, nutritional, and behavioral interventions for the amelioration of oxidative stress- and age-associated diseases and disabilities in humans.

## Introduction

Chronic oxidative stress has been shown to reduce lifespan in many species and lead to accelerated aging [1]–[3]. It has also been reported that oxidative stress is involved in neurodegeneration, sarcopenia and other muscle wasting conditions, which are accompanied by multiple aging symptoms [4]–[6]. Reactive oxygen species (ROS) are generated during normal cellular metabolism, primarily as a result of inefficiencies in the electron transport chain during mitochondrial respiration. Optimally localized levels of ROS serve functionally in the activation of some signal transduction pathways. However, ROS can also cause damaging chemical modifications of macromolecules such as proteins, lipids and DNA, which can in turn contribute to the progression of neurological diseases and neuromuscular disorders including Huntington's disease, Parkinson's disease, Alzheimer's disease, amyotrophic lateral sclerosis, and ataxia telangiectasia [4],[7].

The genetic regulation of the stress and damage response pathways in vertebrates may be more complex than that seen in simple model organisms such as *Drosophila* and *C. elegans*. However, a strong case can be made for repeating the genetic screens performed in these lower organisms, in a vertebrate model, to identify genes regulating oxidative stress. Such analyses have the potential to identify candidate genes related to multiple stress- and age-associated diseases in humans. However, due to the challenges of performing large-scale forward genetic screens in mice, it would be of considerable benefit if the high-throughput screening technology used in simpler organisms (i.e., invertebrates) can be adapted for use in zebrafish (*Danio rerio*), a vertebrate model in which forward genetic screens are routinely performed [8]–[10]. The zebrafish is inexpensive to maintain and has favorable characteristics for experimentation such as a high fecundity, rapid external development, embryonic translucence, and ease of genetic manipulation. In addition, the sequence of the zebrafish genome, while not yet completely annotated, has already revealed a high degree of similarity between fish and human genes.

Thus far, we and two other groups have mainly contributed to establish important baseline information validating the use of zebrafish as a valuable model for aging studies [11]–[16]. We have extensively searched for various biomarkers of aging in zebrafish [17]. However, to faithfully monitor the wide-ranging *in vivo* effects of several stresses on senescence and aging in zebrafish in a high-throughput manner, we required a reliable and easily applicable biomarker that robustly indicates presence of oxidative stress during embryonic development as well as symptoms of aging in adults. One obvious candidate was senescence-associated β-galactosidase (SA-β-gal), a marker of cellular senescence *in vitro* as well as of organismal aging in vertebrates [16], [18]–[21]. Importantly, genes known to cause embryonic senescence can be detected by SA-β-gal in mice [22],[23]. Mounting evidence suggests that the identity of SA-β-gal is in fact the well characterized lysosomal β-galactosidase enzyme, which is most active at a much lower pH, but has some minimal activity at pH 6.0 where it can be detected when abundant [24],[25]. The cellular lysosomal content increases in aging cells due to the accumulation of non-degradable intracellular macromolecules and organelles in autophagic vacuoles [26]. Thus, lysosomal β-galactosidase induction could represent a general adaptive response to cellular senescence. Oxidized protein and lipid by-products that cannot be degraded by lysosomal hydrolases nor be exocytosed accumulate over time in post-mitotic cells, and are not diluted by cell division. One such by-product is lipofuscin, also known as “age pigment” [27]. Lipofuscin is composed of cross-linked protein and lipid residues [28],[29] and is generated by iron-catalyzed oxidative processes as well as by the incomplete degradation of damaged mitochondria [30],[31]. It has previously been demonstrated that both oxidative stress and aging promote lipofuscin accumulation [32].

In our current study, we demonstrate that the level of SA-β-gal is elevated in zebrafish embryos exposed to acute but sub-lethal levels of oxidative stress as well as in aged adults. We then present two genetic screens for mutants in stress responses that might also display altered aging phenotypes. We first examined a collection of retrovirus-mediated insertional mutants for the embryonic induction of SA-β-gal. The screening of these mutants for modified SA-β-gal activity could be performed relatively quickly in the absence of an extrinsic insult and stress, since the homozygous embryos can be identified by their morphology. We found from our results that, of the 306 insertional mutations we examined, at least 11 scored as having significantly elevated SA-β-gal production levels in homozygous mutant embryos, two of which we present in further detail herein. The mutation which resulted in the highest SA-β-gal levels caused an inactivation in a gene encoding the zebrafish homologue of the *Drosophila spinster*, which is responsible for regulation of aging and lifespan in flies and has been implicated in a lysosomal storage function [33],[34]. One of the other mutants inactivated the *telomeric repeat binding factor a (terfa) gene*, a zebrafish homologue of the *telomeric repeat binding factor 2* (*terf2*) gene, which plays prominent roles in telomere protection and telomere-length regulation [35],[36].

For our second screen, we developed a new zebrafish mutant screening protocol based upon N-ethyl-N-nitrosourea (ENU) chemical mutagenesis. We performed a sensitized dominant screen in the zebrafish to detect mutations in the heterozygous state by using a chemical sensitizer (rather than a genetic sensitizer). In our pilot screen using this methodology, we obtained eight mutants in two complementation groups that showed altered SA-β-gal activity in response to oxidative stress. Importantly, adult fish that were heterozygous for several of these mutations also showed premature expression of aging markers/phenotypes, and a shorter lifespan. Our new screening strategy using a senescence-associated biomarker during the embryonic stages in zebrafish provides a new tool for the genetic dissection of vertebrate stress responses and aging mechanisms. Moreover, our initial results strongly suggest that genetic lesions in certain early developmental mechanisms lead to late adult-onset phenotypes with age.

## Results

### SA-β-gal Is a Robust Biomarker of Aging in the Adult Zebrafish

**Figure 1:**
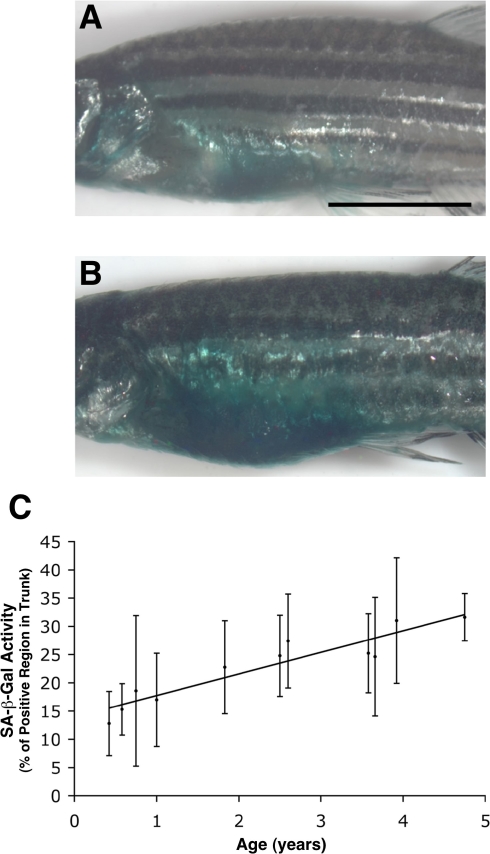
SA-β-gal activity in the trunk skin of the adult zebrafish increases with age. (A, B) Lateral imaging of 5-month (0.42 y) old (A) and 57-month (4.75 y) old (B) whole adult zebrafish stained for SA-β-gal activity. (C) Quantitative analysis of trunk SA-β-gal staining in fish of various ages showing a near-linear increase in SA-β-gal activity with age. Quantitation was done via image analysis using Adobe Photoshop as described in Materials and Methods and documented in the Supporting Information (Figure S1). The numbers of fish at each time point (years) were: 0.42 y (n = 16); 0.58 y (n = 14); 0.75 y (n = 13); 1 y (n = 14); 1.83 y (n = 15); 2.5 y (n = 6); 2.6 y (n = 8); 3.58 y (n = 14); 3.66 y (n = 12); 3.92 y (n = 22); 4.75 y (n = 5). R^2^ = 0.3027. y; year(s) of age. (Scale bar: 0.5 cm.)

To further characterize aging in the adult zebrafish, we have previously examined several potential biological and biochemical markers, including regenerative competence and assays for the oxidative damage of proteins, lipids and DNA [16],[17],[19]. The most reliable and readily detectable age-dependent marker was determined to be a histochemical assay for SA-β-gal activity, which can be quantitatively applied to whole adult zebrafish using X-gal as a substrate at pH 6.0 [16]. In our current experiments, staining for SA-β-gal was found to increase in the skin of zebrafish with age throughout their lifespan (n = 139) (Figure 1A, B), as was previously reported in both humans and zebrafish [16],[18],[19]. To quantitatively examine SA-β-gal levels *in vivo*, we generated high-resolution digital images that enabled us to select stained pixels using image analysis software and to then calculate the percentage of stained pixels out of the net total in each case (Figure S1). Unlike other markers that tended to vary discontinuously with age, we found that SA-β-gal activity increases linearly with age in adult fish ranging in age from 5 to 57 months (Figure 1C).

### SA-β-gal Activity in Zebrafish Embryos Is Responsive to Oxidative Stress

**Figure 2:**
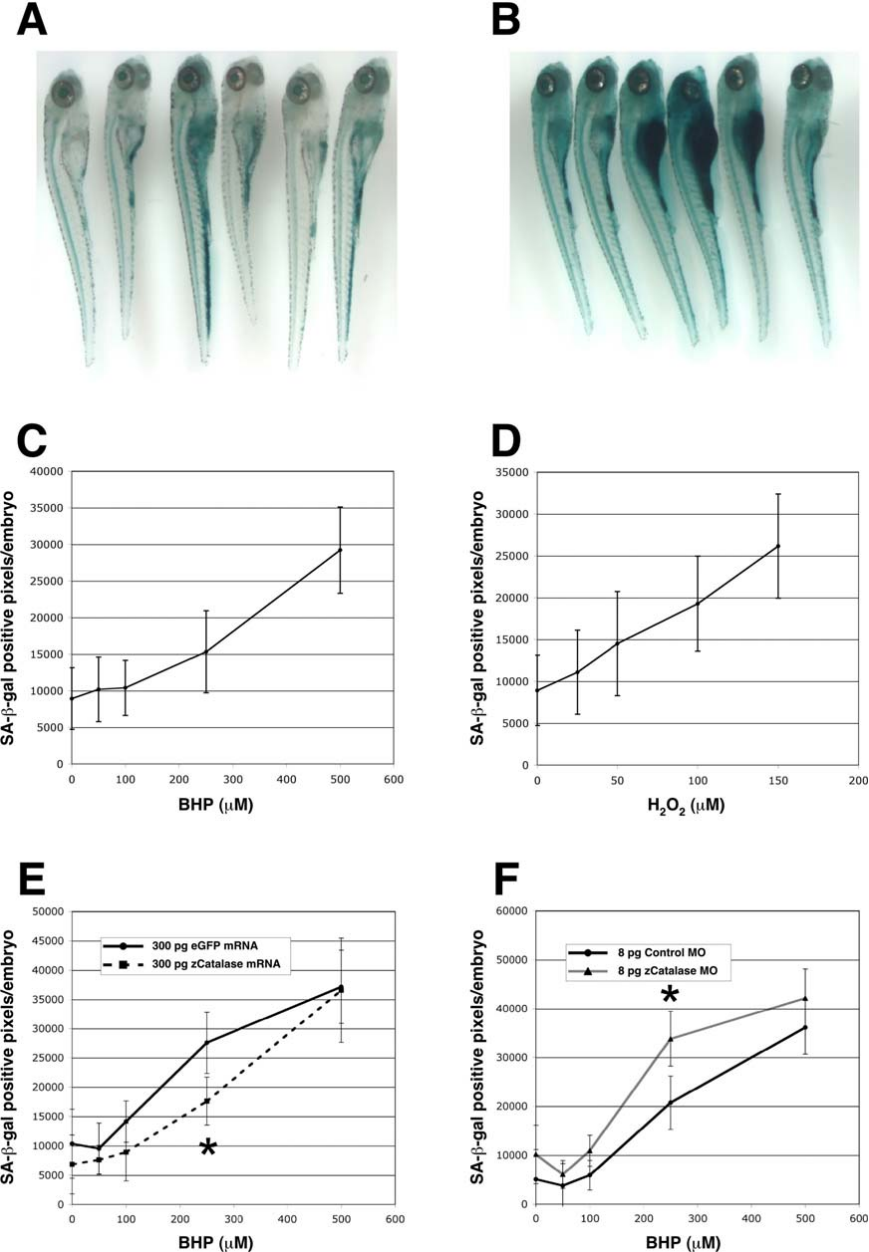
Induction of embryonic SA-β-gal activity by oxidative stress. (A–D) Embryos treated with 500 µM BHP from 6 hpf to 6 dpf (B) have higher SA-β-gal activity than untreated embryos (A). Plots of the colorimetric quantitation of SA-β-gal staining show a near-linear increase of SA-β-gal activity with increased oxidative stress by BHP (C) or hydrogen peroxide (H_2_O_2_) (D). (E) Catalase overexpression can reduce SA-β-gal induction in embryos treated with moderate amounts of oxidative stress. Embryos injected with 300 pg of catalase mRNA at the one cell stage show significantly less SA-β-gal activity at 6 dpf than those injected with a control mRNA following incubation with 350 µM BHP from 6 hpf to 6 dpf. (F) Knockdown of endogenous catalase sensitizes embryos to oxidative stress-dependent SA-β-gal induction. Embryos injected with 8 ng of an antisense morpholino (MO) for catalase show a significant increase in SA-β-gal activity at 6 dpf when stressed with 350 µM BHP from 6 hpf to 6 dpf. *P<0.05 (Student t-test).

We hoped to avoid the need to screen for aging mutants using actual lifespan analyses if we instead screened embryos for mutations that alter the expression of aging markers in response to oxidative stress [17]. For such an approach to succeed, we surmised that the chosen biomarker must respond both to aging in adults and to stress responses during embryonic development. Hence, we tested whether the SA-β-gal assay would also respond to oxidative stress in embryos treated with ROS such as hydrogen peroxide (H_2_O_2_) or *tert*-butyl hydroperoxide (BHP) (Figure 2A–D). Several different doses of these peroxides were used over a developmentally long period to better simulate long-term chronic oxidative stress. An experimental endpoint at 6 days post fertilization (dpf) was chosen to avoid potential spurious effects of caloric restriction or other nutritional deficiencies, as this is the point in larval development at which the supportive yolk has been consumed and the fish begin to eat and rely on oral intake nutrition. The LD_50_ values for H_2_O_2_ and BHP were measured at approximately 300 µM and 1 mM, respectively, under these assay conditions. At sub-lethal doses of peroxides, SA-β-gal levels increased in a roughly linear fashion with increasing concentrations of H_2_O_2_ and BHP to the maximum tolerated doses of 150 µM and 500 µM, respectively. Compared with the untreated controls (n = 50) (Figure 2A), zebrafish embryos treated with either 150 µM of hydrogen peroxide (n = 50) or 500 µM of BHP (n = 50) displayed an approximately 3-fold increase in SA-β-gal staining intensity following six days of development (Figure 2B–D). These results suggested that SA-β-gal-based screens of chemically or genetically stressed embryos could indeed be used to identify senescence-related mutants in zebrafish.

### Modulation of Oxidative Stress-Induced Embryonic SA-β-gal in Zebrafish by Reverse Genetic Manipulation

To test whether the induction of SA-β-gal activity in zebrafish embryos that have been exposed to oxidative stress occurs in a similar manner to that reported in other organisms, we performed genetic manipulations of a ROS detoxification enzyme *in vivo*. A number of studies in a variety of species have shown that both catalase and glutathione-peroxidase are responsible for antioxidant protection by limiting the accumulation of hydrogen peroxide [2],[3],[37],[38]. To ascertain the potential importance of catalase in protecting zebrafish embryos from oxidative stress-induced senescence, we altered the expression levels of this enzyme in stressed embryos and measured the effects of this upon SA-β-gal activity. Embryos overexpressing zebrafish catalase were generated by the injection of 300 pg of mRNA encoding this enzyme at the one-cell stage (n = 50). This resulted in a reduction in both hydrogen peroxide- and BHP-induced SA-β-gal activity, compared with control GFP mRNA injections (n = 50) (Figure 2E and data not shown). BHP was used as the oxidative agent throughout the later stages of this study as it is more stable than hydrogen peroxide and produces less variable stress responses. The most dramatic rescue effects were observed when intermediate concentrations of BHP were used in these catalase experiments.

We additionally tested if a reduction in the catalase expression levels would enhance the induction of SA-β-gal activity by oxidative stress. To this end, we injected embryos with an antisense morpholino oligonucleotide (MO) targeting zebrafish catalase and, indeed, observed a marked enhancement in their susceptibility to elevated SA-β-gal activity following exposure to oxidative stress (Figure 2F). The greatest effects were again observed when intermediate concentrations of BHP were used. These results confirmed that the manipulation of a single gene can modulate the SA-β-gal activity levels induced by oxidative stress in zebrafish embryos and prompted us to pursue a genetic screening project to uncover potential aging mutants.

### Potent Induction of Embryonic SA-β-gal Activity in Zebrafish *spinster* and *terf2* Homologue Mutants

We hypothesized that a loss-of-function (or even partial loss-of-function/decrease-of-function) mutation in certain genes may induce specific stress conditions in mutant embryos. To identify potential aging mutants, we first screened for mutants with an altered production of the stress response marker SA-β-gal in an established zebrafish mutant collection generated by retrovirus-insertional mutagenesis [39]. Currently, the Hopkins' insertional mutant collection contains more than 500 recessive mutants with morphological embryonic phenotypes, which include mutations in 335 different identified genes [10],[40],[41]. We screened unstressed homozygous embryos derived from incrossed heterozygotes from 306 of these lines for SA-β-gal expression at 3.5–5 dpf, depending upon the onset of the morphological phenotype.

**Figure 3:**
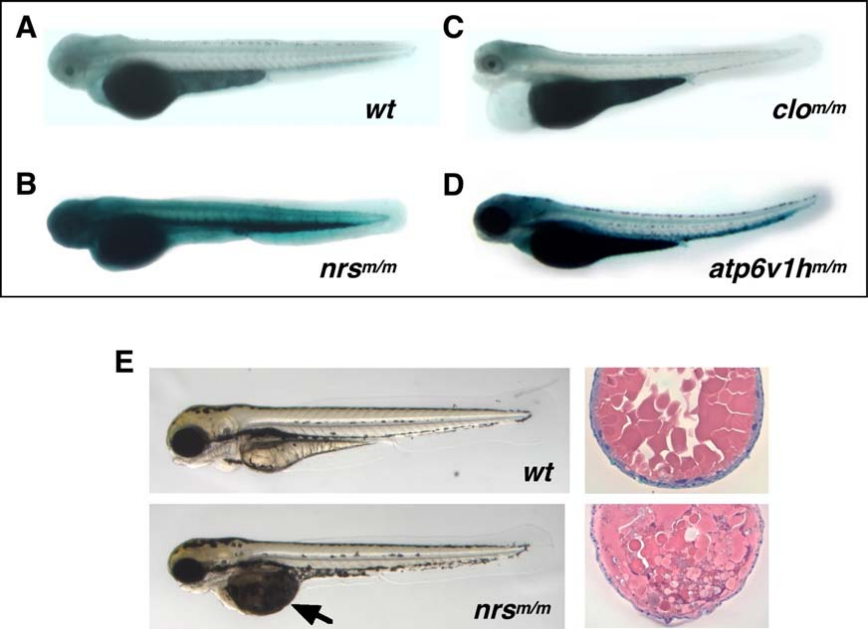
Mutant *nrs* zebrafish show extremely high SA-β-gal activity and display both yolk and muscle phenotypes. 3.5-day old (3.5 dpf) homozygous *nrs* ^m/m (hi891/hi891)^ zebrafish embryos show extremely high SA-β-gal activity (B) compared with wild-type embryos (A) (with PTU). The *atp6v1h* ^m/m (hi923/hi923)^ mutant shown in (D) is another variant identified as having significantly higher SA-β-gal activity. Most other early embryonic lethal mutants derived from either insertional mutagenesis (as shown in Figure S4) and chemical mutagenesis (e.g., *clo* ^m39/m39^ which is shown in (C)) show no higher (or indistinguishable) SA-β-gal activity than wild-type siblings at any time during development. (E) Yolk opaque phenotypes can be observed in homozygous *nrs* ^m/m^ embryos at 3.5 dpf (earliest detection at around 2.5 dpf; lower left panel), compared with wild-type embryos (upper left panel). Also shown is a comparison between the H&E staining of transverse sections of the yolk part of *nrs* ^m/m^ embryos at 3.5 dpf (lower right panel) and wild-type embryos (upper right).

In general, the levels of SA-β-gal seen in the homozygous mutants were low, with only 11 mutants clearly scoring robustly higher than wild-type background activity (Figure 3; Figures S3 and S4) (Table 1). It should be noted also that since all of the 306 mutations screened are ultimately homozygous lethal, these data indicate that SA-β-gal production is not a general result of embryonic death. Figure S4 shows several examples of embryonic lethal mutants whose SA-β-gal levels are no higher than (or indistinguishable from) their wild-type siblings despite varying amounts of cell death. Similarly, the *cloche* (*clo*) mutant, which has no circulatory system did not show detectable SA-β-gal induction above background activity (n = 54) (Figure 3C). However, for 11 of the lines, the mutant embryos showed significantly stronger SA-β-gal staining than their wild-type siblings. For example, as shown in Figure 3D, an insertional mutation in the *atp6v1h* gene encoding the V1 subunit H component of vacuolar ATPase (v-ATPase), a multi-subunit enzyme that mediates the acidification of eukaryotic intracellular organelles, is one of the 11 mutants identified that showed robust levels of SA-β-gal induction (n = 45).

**Table 1:** Retrovirus-insertional mutants having higher SA-β-gal activity.

Family* (and other alleles)	Zebrafish Gene*	Phenotypic Description*	SA-β-Gal Activity (in homozygous embryos)
Wild-type Control	________	__________	Mean: 5859
			SD: 1163
			N: 49
hi891	spinster homolog	day 2: very dark/rotting yolk with thin or no extension;	Mean: 11127
		day 5: thin, necrotic, big edema, dead or dying	SD: 2418
			N: 38
hi1182	telomere binding protein 2	day 2: central nervous system (cns) necrosis;	Mean: 8993
hi3678**		day 3: small head and eyes,	SD: 1776
		day 5: small head, very small eyes, sometimes do inflate swim bladder	N: 49
hi4201b	cdc39-like	day 3: small head and eye;	Mean: 9367
		day 5: small head and eye, underdeveloped liver/gut, a little pericardial edema and edema around eyes or head	SD: 1863
			N: 48
hi447	denticleless homolog	day 1: cns necrosis, flat head, thin yolk extension;	Mean: 8974
hi3627**		day 2 very bad necrosis; most dead by day 5 (see Sansam et al., Genes Dev 20, 3117–29, 2001)	SD: 1924
			N: 49
hi1520	enthoprotin	day 1/2: sometimes slightly smaller head and eyes, sometimes smaller body size (highly variable in this respect);	Mean: 7925
		day 3–5: skin looks bumpy, often shorter body, sometimes small pericardial edema;	SD: 1667
		day 5: also underdeveloped liver/gut, unconsumed yolk	N: 44
hi2718b	RNA polymerase II subunit D	day 1: cns necrosis, flat head;	Mean: 9164
		day 2: very bad necrosis; usually dead by day 5	SD: 1765
			N: 49
hi3685	RNA polymerase II subunit G	day 1: cns necrosis, slightly flat head;	Mean: 8927
		day 2: very small head and eye, thin body, pale, pericardial edema denting yolk, thin yolk extension;	SD: 1808
		day 5: very small head and eyes, pericardial edema, thin/wasted body, often dead	N: 45
hi1113a**	chromosome adhesion protein	day 2: cns necrosis, rounder dark yolk, body may be bent dorsally; slater days necrosis worsens through head and body, dead or very necrotic by day 5	Mean: 9105
hi2068	SMC1-like		SD: 1524
hi2327			N: 43
hi3821			
hi229**	smoothened	day 1: U-shaped somites, mildly cyclopic;	Mean: 9159
hi1640		day 2: smaller eyes, too close together, pc edema denting yolk, which is grey and bloated, body thin and curled ventrally with U-shaped somites, absence of lateral floor plate and secondary motoneurons;	SD: 1703
hi2329b		usually dead by day 5 (see Chen, et al.,Development 128, 2385–96, 2001)	N: 48
hi923	v-ATPase SDF/54kD	day 2: no/less body pigment;	Mean: 8985
	subunit	day 5: less body pigment, small head and eyes, underdeveloped liver/gut, sometimes some pericardial edema	SD: 1564
			N: 45
hi1207	v-ATPase 16kD proteolipid	day 2: no pigment and a little cns necrosis;	Mean: 8785
	subunit	day 5: less pigment, also small head and eyes, underdeveloped liver/gut, sometimes bent and/or dying	SD: 2101
			N: 49

*: All the information is available at 〈http://web.mit.edu/hopkins/index.html〉.

**: The allele quantitatively estimated for SA-β-gal activity shown in this table.

*P*-value of SA-β-gal activity is *P*<0.0001 in every mutant listed above (Student's t-test).

We chose to study two out of 11 of the insertional mutants in more detail based upon previous knowledge about the mutated genes in other organisms. Of these insertional mutants, the highest SA-β-gal activity was found to be associated with an insertion in the gene denoted “*not really started*” (*nrs*) (currently denoted as zebrafish *spinster homolog 1, spns1*) (*hi891*) (*nrs* mutant, n = 135; wild type, n = 185) (Figure 3B) [42]. The *nrs* ^m/m^ homozygotes die by 4 dpf and show a substantial accumulation of an opaque substance in the yolk (Figure 3E, indicated by a black arrow in the left lower panel). Furthermore, the *nrs* gene has been identified as the zebrafish homologue 1 (*spns1*) of the *Drosophila spinster* (*spin*) gene. A *Drosophila* partial loss-of-function (hypomorphic) mutant for the spinster gene accumulates lipofuscin granules in the central nervous system, accompanied by neurodegeneration and abnormal ovary development. Notably, *Drosophila* hypomorphic *spin* mutants also have a shortened lifespan [33].

**Figure 4:**
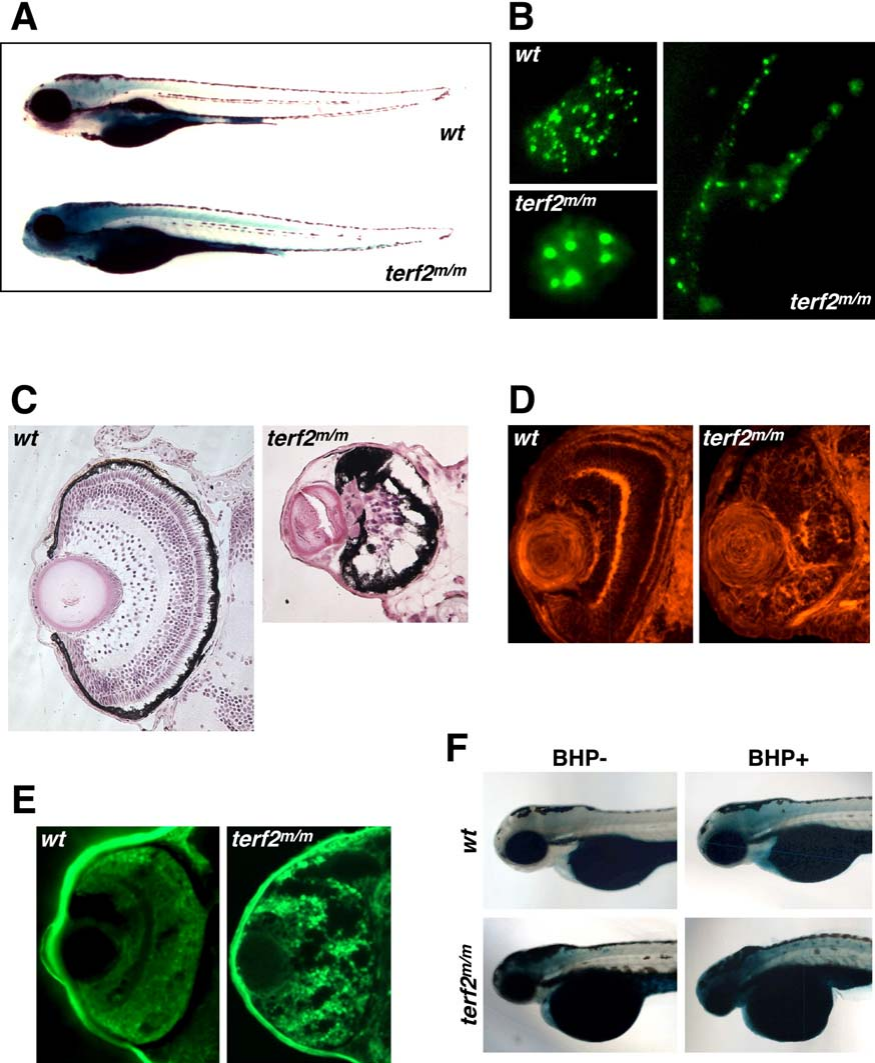
Mutant *terf2* animals with high SA-β-gal activity and retinal neurodegenerative phenotypes. (A) 4.5-day old (4.5 dpf) homozygous *terf2* ^m/m^ zebrafish larvae show high SA-β-gal activity, particularly in the brain and spinal cord, having the small eyes and head (lower image), compared with wild-type (upper image). (B) Abnormally enlarged telomere speckles (lower left panel) and aberrant nuclear shapes (right panel) can be observed in homozygous *terf2* ^m/m^ embryos, compared with normal telomere speckles in a nuclear of the wild-type embryo (upper left panel) at 5 dpf. (C) H&E staining of transverse sections through the retinas of homozygous *terf2* ^m/m^ (right panel) and wild-type zebrafish embryos (left panel) at 5 dpf. (D) Embryonic zebrafish retinas were stained with phalloidin to visualize the actin filaments in the plexiform layers, which revealed obvious structural defects in the homozygous *terf2* ^m/m^ mutant (right panel) compared with a wild-type sibling (left panel) at 3 dpf. (E) Neurodegeneration in the retina was histologically detected by performing Fluoro-Jade B staining in homozygous *terf2* ^m/m^ mutant (right panel), but was not evident in the wild-type embryo (left panel) at 2 dpf. (F) Homozygous *terf2* mutant embryos and wild-type controls were exposed to 350 µM BHP from 6 hpf to 4 dpf. Enhanced SA-β-gal staining with a more severe morphology in eyes and heads are observed in BHP-treated *terf2* mutant embryos at 4 dpf (lower right panel).

The other mutant which we focused on exhibited an insertion in the “*telomere repeat binding factor a*” (*terfa*) gene. The mutant lines *hi3678* (n = 155) and *hi1182* (n = 120) (Figure 4A, lower image for *hi3678* and Figure S2B, lower right panels for *hi1182*) had significantly higher SA-β-gal activity compared with wild-type controls (n = 100 for each) (Figure 4A, upper image; Figure S2B, lower left panel). The *terfa* gene is a zebrafish homologue of the human *terf2* gene which encodes the telomeric repeat binding factor 2 protein (TRF2). Due to the varied nomenclature for *terfa* in other species, we denote the zebrafish gene as *terf2* hereafter. TRF2 has an essential role in telomere end protection and t-loop formation [35],[43],[44]. Moreover, the disruption of endogenous TRF2 function in human cells by expressing dominant-negative forms of this protein markedly increases the rate of telomere end-to-end fusions and cellular senescence [36]. A deletion of the *terf2* gene in mouse embryonic fibroblasts also results in a senescence-like arrest and SA-β-gal induction [45]. Hence, the SA-β-gal induction that we see in our zebrafish *terf2* mutant embryos is consistent with the established biological role of this gene and the results of previous studies in other organisms.

Telomeres of homozygous *terf2* embryos were visualized by cross mating with transgenic fish expressing a green fluorescence protein (GFP)-tagged human TRF1/Pin2 fusion protein [46]. While many telomere speckles were evident in the wild-type background (n = 20) (Figure 4B, upper left panel), we observed enlarged telomere speckles and abnormal nuclear shapes in *terf2* ^m/m^ fish embryos (n = 24) (Figure 4B, lower left and right panels), which are likely to reflect telomere end-to-end fusions and impaired chromosome integrity. Moreover, homozygous *terf2* ^m/m^ mutant zebrafish embryos showed aggressive neurodegenerative phenotypes in the eye, brain, and spinal cord (n = 32) (Figure 4C, right panel; Figure 7W–Y), compared with wild type (n = 10) (Figure 4C, left panel; Figure 7T–V). In contrast to normal retinal development in wild-type embryos (n = 5) (Figure 4D, left panel), embryonic retinas stained with phalloidin in order to visualize actin filaments in plexiform layers revealed obvious structural defects in *terf2* ^m/m^ mutants (n = 10) (Figure 4D, right panel). Neurodegeneration in the retina was also detected histologically by performing Fluoro-Jade B staining of *terf2* ^m/m^ embryos (n = 10) (Figure 4E, right panel), compared with normal wild-type retinas (n = 5) (Figure 4E, left panel). Significantly, the neural phenotypes associated with a *terf2* mutation appear to be consistent with the recent observations of mammalian TRF2 function reported in neural cells *in vitro* [47],[48]. While we did not examine other mutant lines at this level of detail, it was clear that some of the other insertional mutants with elevated SA-β-gal levels also exhibited widespread cell death in the central nervous system and eyes (Table 1).

To further examine the SA-β-gal induction caused by disruptions of the *nrs* and *terf*2 genes, we knocked down the translation of their respective mRNAs using MOs that target the start codon of each gene. Injection of a *nrs* MO at the single- or two-cell stage resulted in an exact phenocopy of the *nrs* mutant embryos which manifested obvious yolk opacity. Upon SA-β-gal staining, an extremely high level of induction was observed in the *nrs* morphants (n = 116 of 120; 97%), identical to the SA-β-gal levels in the *nrs* mutants (Figure S2A). In contrast, the control embryos did not show any significant SA-β-gal activity (n = 150). We also injected a *terf2* MO into zebrafish embryos, and observed robust SA-β-gal induction (n = 191 of 200; 95.5%), and a relatively moderate morphological phenotype similar to the *terfa* ^hi1182/hi1182^ allele, that has a weaker phenotype than the insertional mutant (*terfa* ^hi3678/hi3678^) used above (Figure S2B). This is consistent with the higher residual levels of *terf2* mRNA in the morphants and in the *hi1182* mutants harboring an insertion in the first intron of the *terf2* gene which allows for the production of some wild type transcript, in contrast to the *hi3678* allele that has the insertion in the first exon of the *terf2* gene (http://web.mit.edu/hopkins/insertion20sites/1182.htm).

We additionally exposed *nrs* and *terf*2 mutant embryos to oxidative stress by BHP treatment. The homozygous *terf*2^m/m^ (*terfa* ^hi3678/hi3678^) mutants (n = 50), but not the heterozygotes, clearly show enhanced induction of SA-β-gal activity with a more severe morphology in the eyes and heads (Figure 4F), whereas no significant difference was observed in the *nrs* mutant animals of either homozygous or heterozygous backgrounds (data not shown). Taken together, the outcomes from our current screen of 306 lines from the Hopkins' insertional mutant collection serve as a proof of concept for our strategy and the first success in our novel approach to identify potential aging-related genes by examining the senescence-associated biomarker in zebrafish embryos.

### A Screen for Zebrafish Mutants Showing Stress-Induced Premature Embryonic Senescence

Having established that SA-β-gal is induced by oxidative stress caused by BHP treatment, we next performed a new screen for ENU mutant zebrafish which displayed phenotypic alterations arising from genetic mutations in stress response mechanisms. We crossed individual F_1_ mutant males with wild-type females to produce clutches of F_2_ embryos, each of which was heterozygous at many loci. By using BHP as a chemical sensitizer, we hoped to identify heterozygous mutants with an altered response to oxidative stress; that is we expected the chemical sensitizer to induce haploinsufficiency in many of the potential target genes. We have denoted this methodology ‘CASH’ (Chemically Assisted Screening in Heterozygotes).

**Figure 5:**
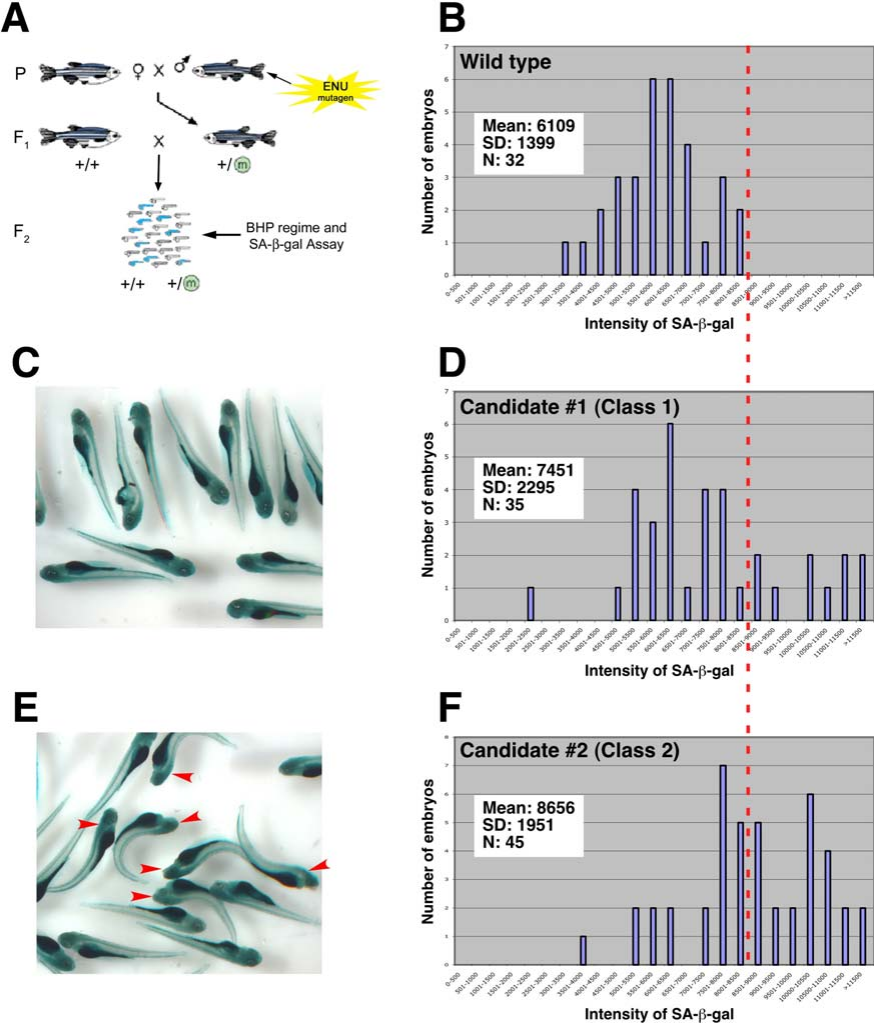
CASH screening methodology and mutant candidate identification. (A) Schematic representation of mutagenized wild-type male zebrafish bred with wild-type females. Males from the resultant F_1_ generation are raised and outcrossed with wild-type females. The resultant embryo clutches will be 50% wild type and 50% heterozygous with respect to a mutation generated in the parental (P) gametes. These F_2_ embryos are treated with moderate levels of BHP and stained for SA-β-gal activity (C). Quantitation of the staining levels is then performed. Embryo counts are grouped into 500-pixel intensity cohorts and plotted. (B) Wild-type embryos show a tight Gaussian distribution of staining intensity. Significantly right-shifted distributions (dotted red line in D) identify Class 1 candidates in terms of their oxidative stress sensitivity (D). Class 2 mutant candidates are identified if direct phenotypic abnormalities (red arrows) exist in approximately 50% of the embryos of clutches only in the presence of exogenous oxidative stress (an example is shown in E and F), with significantly right-shifted distributions (dotted red line in F).

We treated 50 embryos from the resulting clutches with 350 µM BHP from 6 hours post fertilization (hpf) to 6 dpf. In each clutch of embryos, half of the clutch would be heterozygous for any mutant allele, and half would be wild type for that allele. Thus, an F_1_ male carrying a mutation that alters sensitivity to oxidative stress would produce clutches wherein half the embryos show altered induction of SA-β-gal activity (Figure 5A). We divided SA-β-gal staining intensity in the F_2_ embryos into discrete quantitative ranges and measured how many embryos fell into each staining intensity range. When we performed this analysis on wild-type embryos, the result was a tight Gaussian distribution (Figure 5B). When we looked at our candidate mutant clutches, in most cases their staining distributions appeared similar to wild type. However, occasionally, nearly half of the embryos were darker than the others and the distributions appeared abnormal (see a dotted red line throughout Figure 5B, 5D, and 5F). The F_1_ fathers of these clutches were potential carriers of mutations that either enhanced SA-β-gal activity. These are what we will refer to as ‘Class 1’ mutant candidates (Figure 5C and 5D; n = 35 for this tested candidate; *P*<0.01, Student t-test). We also observed clutches of another class of mutants in which approximately half of the embryos showed a clear morphological abnormality in the presence of BHP (Figure 5E and 5F; n = 45 for this tested candidate; *P*<0.001, Student t-test). However, when we repeated these outcrosses without exogenous oxidative stress, the resultant clutches appeared morphologically normal. These clutches comprise what we will refer to as ‘Class 2’ mutant candidates. We performed an initial screen in 150 F_1_ mutagenized genomes, and obtained 8 candidate mutants that bred in a consistent recessive Mendelian fashion through to the F_4_ generation. Six of the mutants were from Class 1 and two were from Class 2.

### Homozygous Phenotypes of Oxidative Stress-Sensitive Zebrafish Mutants

**Figure 6:**
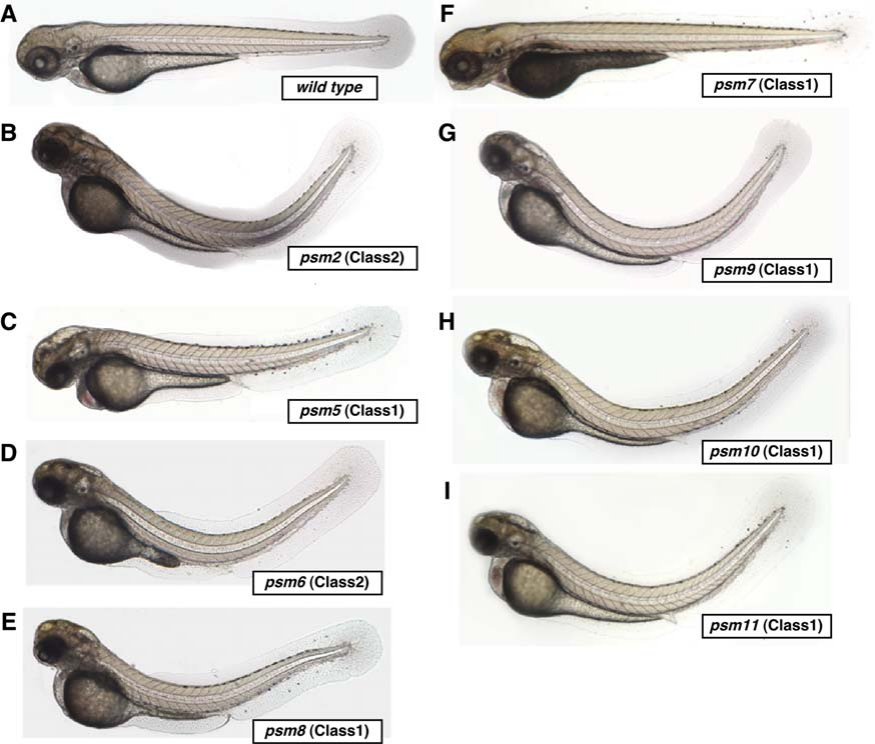
Homozygous *psm* mutant zebrafish embryos and their respective classes. Shown are a wild type embryo (A), and *psm2* (B), *psm5* (C), *psm6* (D), *psm8* (E), *psm7* (F), *psm9* (G), *psm10* (H), and *psm11* (I) mutant embryos (with PTU). Neurodegenerative mutants (*psm2*, *5*, *6*, *8*, *9*, *10*, *11*) have opaque regions in the head and obvious dorsal curvature by 3.5 dpf as homozygotes. The homozygous muscle atrophy mutant *psm7* is accompanied by an opaque yolk and a slightly protruding jaw. Class 1 mutants (*psm5*, *7*, *8*, *9*, *10* and *11*) were revealed by our oxidative stress CASH screen as candidates that displayed high SA-β-gal activity in heterozygotes when stressed with 350 µM BHP from 6 hpf to 6 dpf. Class 2 mutants (*psm2* and *6*) showed obvious SA-β-gal activity, but morphologically abnormal phenotypes in heterozygotes when stressed with 350 µM BHP from 6 hpf to 6 dpf in the CASH screen.

When we incrossed F_2_ siblings (n>30) of each peroxide-sensitive mutant (*psm*) line, we observed the homozygous phenotypes seen in Figure 6 in about 25% of the embryos of each clutch from about 1 out of every 4 incrosses. This is consistent with the expected Mendelian segregation of a recessive trait. For each *psm* mutant, over 2 generations and at least 20 fish per generation the fish that transmitted the heterozygous *psm* phenotype with high SA-β-gal activity also transmitted the recessive morphological phenotype. Seven of the phenotypes were very similar (*psm2, 5, 6, 8, 9, 10, 11*), with evidence of a moderate dorsal curvature of the trunk, and showed pronounced levels of cell death which was clearly apparent in the brain beginning at 48 hpf (n = 50 for each mutant) (five of these were Class 1 mutants and two were Class 2 mutants). These homozygous mutations were embryonic lethal, with death occurring at around 6 dpf. The remaining *psm7* mutant (Class 1) developed a minor protrusion of the jaw and had an opaque yolk that first became apparent at 4 dpf (n = 50), subsequently dying at around 7 dpf.

**Figure 7:**
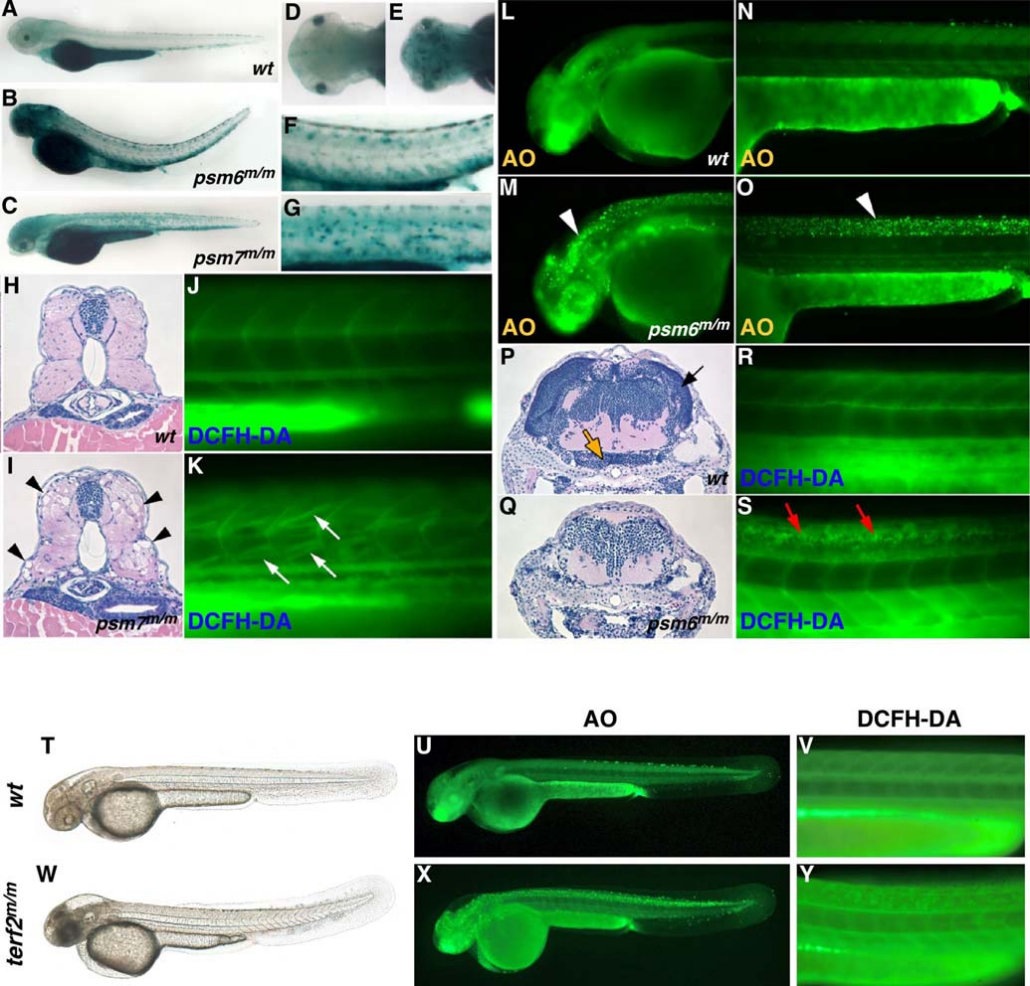
Senescence, cell death and ROS generation in homozygous *psm* zebrafish mutants in the absence of oxidative stress. SA-β-gal activity was found to be high throughout the brain and neural tube in neurodegenerative zebrafish mutants (B and the magnified trunk region in F) [*psm6* ^m/m^ is shown] compared with wild-type embryos (A) at 3.5 dpf (with PTU). (D, E) Dorsal views of the head of wild-type and *psm6* ^m/m^ embryos respectively. (G) Neurodegenerative mutant embryos have high levels of acridine orange (AO) staining (white arrowheads) in the brain (M) and neural tube (O), compared with wild-type embryos (L, N) at 2 dpf. H&E staining of transverse sections of the head of 5-day old (5 dpf) larvae reveals a reduction in the number of neuronal nuclei and absence of brain structures in neurodegenerative mutants (Q), compared with wild-type embryos (arrows in P which indicate the tectum opticum [black arrow] and caudal hypothalamus [orange arrow]). DCFH-DA staining indicates high ROS generation in the neural tube of neurodegenerative mutants (red arrows in S) compared with wild-type embryos (R) at 3.5 dpf. The muscle atrophy mutant [*psm7* ^m/m^ is shown] is characterized by punctate SA-β-gal activity in the trunk (C and magnified trunk region in G). H&E staining of transverse sections of the trunks of 4-day old (4 dpf) *psm7* ^m/m^ larvae reveals the loss of muscle fibers (black arrowheads in I), compared with wild-type embryos (H). DCFH-DA staining reveals the generation of ROS in skeletal muscle in the absence of exogenous oxidative stress in *psm7* ^m/m^ embryos (white arrows in K) compared with wild-type embryos (J) at 3.5 dpf. Morphology of *terf2* ^m/m^ mutant embryo was compared with that of wild-type sibling (T, W) at 2 dpf (with PTU). *terf2* ^m/m^ mutant embryos have high levels of acridine orange (AO) staining (X) in the brain and neural tube, compared with wild-type embryos (U) at 2 dpf. DCFH-DA staining indicates high ROS generation in the neural tube of *terf2* ^m/m^ mutant (Y) compared with wild-type embryos (V) at 3.5 dpf.

We proceeded to examine the homozygous phenotype of each *psm* mutation more closely. All F_3_ homozygous embryos (n = 50) with abnormal phenotypes were also found to have higher SA-β-gal staining than the wild-type controls (n = 20) in the absence of any exogenous oxidative stress (Figure S5). Punctate SA-β-gal staining was seen throughout the central nervous system in each of the seven mutants we identified (*psm2, 5, 6, 8, 9, 10, 11*), which showed brain abnormalities and dorsal curvatures (n = 25 for each mutant) (Figure 7B, 7E and 7F; *psm6* mutant is shown). Acridine orange (AO), which stains dying cells, produced very intense signals throughout the neural tube and brain tissues of these mutants between 36 and 72 hpf (n = 50 for each mutant at each time point), indicating massive cell death (Figure 7M and 7O; *psm6*). *In vivo* staining of the neurodegenerative mutants at 3.5 dpf with dichlorofluorescein diacetate (DCFH-DA), an indicator of ROS, revealed the presence of high levels of ROS in the neural tube, specifically in the dorsal half (n = 20 for each mutant) (Figure 7S; *psm6*). Interestingly, these phenomena were also true of homozygous *terf2* ^m/m^ mutant embryos that showed high levels of ROS in the neural tube at 3.5 dpf (Figure 7Y), and demonstrated positive AO-staining indicating cell death also at 2 dpf (Figure 7X). Histological analysis of these embryos at 2 and 3 dpf indicated evident abnormalities around the regions of the brain, neural tube and eyes where the accumulation of neuronal cell death products (data not shown). At 4 and 5 dpf, further histological examinations revealed that the brain and neural tubes of the *psm* mutants were considerably smaller than those of wild types and contained fewer neuronal nuclei (Figures 7P; wild type and 7Q; *psm6*).

The mutant showing yolk opacity (*psm7*) also showed mottled SA-β-gal staining throughout the muscle in the trunk (n = 71) (Figure 7C and 7G). Histological sections of mutant animals at 4 dpf revealed the absence of muscle fibers throughout the myotomes (n = 35) (Figure 7I), suggesting muscular degeneration. However, this phenotype does not appear to be the result of defective muscle fiber attachment as reported in another dystrophy-like mutant (Bassett et al., 2003), since whole-mount *in situ* histochemical analysis of dystrophin expression appeared to be normal (n = 20) (Figure S6), indicating that the fiber loss is not likely related to dystrophin-mediated fiber adhesion. In addition, DCFH-DA staining of the *psm7* homozygotes at 3.5 dpf showed high ROS production in many individual muscle fibers (n = 45) (Figure 7K).

It is noteworthy that all of the seven neurodegenerative mutants (*psm2, 5, 6, 8, 9, 10, 11*) were in the same complementation group while the mutant showing muscle degeneration (*psm7*) was not linked to any of the neural phenotype mutants (Table S1). Moreover, each of the *psm* mutants complemented both the *nrs* and *terf2* mutations (Table S1). Thus it is possible that this pilot ENU screen has recovered mutations in only 2 genes, each of which are distinct from the *nrs* and *terf2* genes.

### Adult Zebrafish Heterozygous for *nrs*, *terf2*, and *psm* Mutations Exhibit Elevated Levels of Biomarkers and Phenotypes Associated with the Onset of Aging

**Figure 8:**
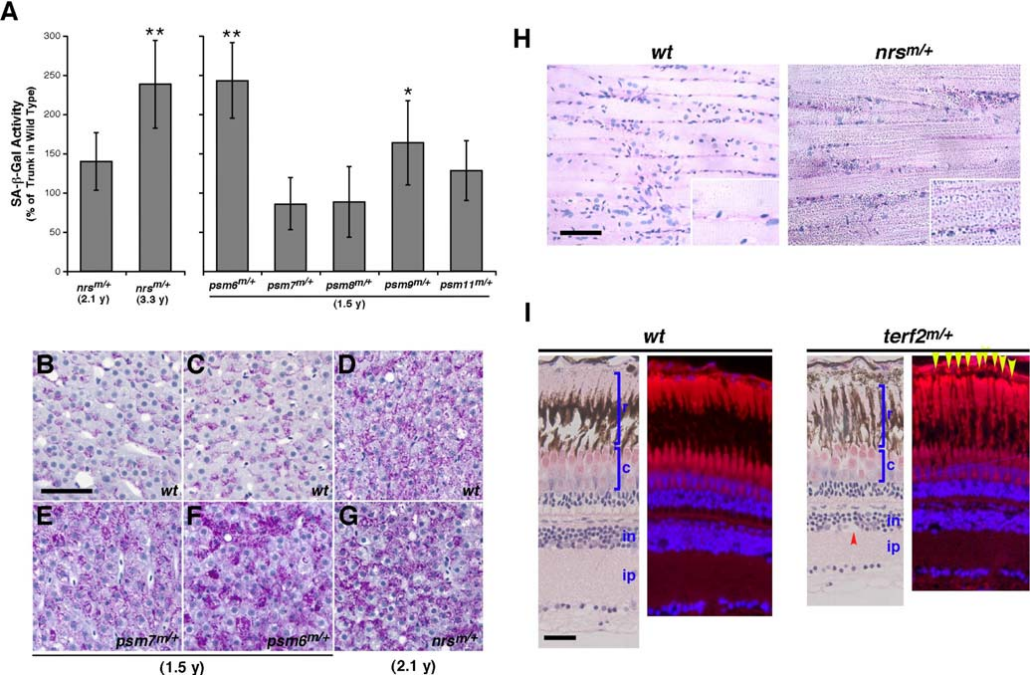
Heterozygous mutant zebrafish with increased SA-β-gal activity and elevated levels of aging biomarkers in adults. (A) The heterozygous *nrs* ^m/+^ mutants showed significantly increased SA-β-gal activity in the skin compared with their wild-type siblings at 3.3 y, but not before 2.1 y (right side graph). Two *psm* mutants (*psm6 and psm9*) also showed significantly increased SA-β-gal activity in the skin as heterozygotes, compared with their wild-type sibling counterparts at just 1.5 y (left side graph). *P<0.05; **P<0.01 (Student t-test). (B–G) Shown here are liver sections from a 1.5 y *psm7* ^m/+^ heterozygote and a wild-type sibling (E and B, respectively), and a 1.5 y *psm6* ^m/+^ heterozygote and a wild-type sibling (F and C, respectively). All of the heterozygous male *psm6* ^m/+^ (n = 8) and *psm7* ^m/+^ (n = 6) mutants analyzed show increased lipofuscin accrual in the liver. Representative liver sections from a 2.1 y *nrs* ^m/+^ heterozygote and a wild-type sibling are also shown (G and D, respectively) (n = 5 for each). (H) Heterozygous aged *nrs* ^m/+^ mutants show increased lipofuscin accrual in the skeletal muscle (n = 8 for mutants; n = 5 for wild-type siblings). Longitudinal trunk muscle sections from a 33-month (2.8 y) *nrs* ^m/+^ heterozygote mutant and a wild-type sibling are shown (left and right panels, respectively), with enlarged images included as insets. (I) Heterozygous and older (23-month old) *terf2* ^m/+^ mutants show a decreased thickness of the retina, particularly in the photoreceptor layer and inner plexiform layer, compared with age-matched wild-type siblings. Heterozygous older (23-month old) *terf2* ^m/+^ mutants show increased drusen-like accruals (yellow arrows) with autofluorescence surrounding the RPE area and both more and larger empty space(s) (red arrow) in the inner nuclear layer when compared with an age-matched wild-type sibling. (Scale bar: 100 µm.)

We wondered whether there might be long-term (‘aging’) effects of heterozygosity for the genes identified in our screens stemming from the associated embryonic alterations in senescence markers/phenotypes. We thus measured the premature aging marker levels and pathohistological phenotypes in heterozygous fish as they aged. Two of the 5 tested heterozygous *psm* mutant lines (*psm6*, n = 8 and *psm9*, n = 8) showed significantly higher levels of SA-β-gal activity in the skin at just 1.5 years of age (18 months) compared with their wild-type siblings (n = 10 for each group) (Figure 8A). In contrast, a heterozygous *nrs* ^m/+^ mutant (n = 12) showed only a modest increase in skin SA-β-gal activity at 2 years but showed a high induction of SA-β-gal at 3.3 years of age (40 months), compared with wild-type siblings (n = 15) (Figure 8A).

In addition to SA-β-gal activity, lipofuscin is often considered to be a hallmark of aging, showing an accumulation rate that correlates with longevity in some tissues [31],[49]. We reported previously that wild-type adult zebrafish are refractory to the accumulation of lipofuscin in muscle by 2 years of age [19]. In our current study we found that there is still no detectable lipofuscin accumulation in wild-type sibling fish (n = 15) by 2.8 years (33 months) of age, but that heterozygous *nrs* ^m/+^ mutant fish (n = 10) had accumulated a great deal of lipofuscin in the skeletal muscle by this time (Figure 8H). On the other hand, in terms of the liver histology of the wild-type zebrafish, lipofuscin accrual changed dramatically with age (Figure S7A), similar to that reported in mice [50]. Notably also, we found in our current analyses that male *psm6* (n = 10) and *psm7* (n = 10) heterozygous animals, as well as the male *nrs* ^m/+^ (n = 8) heterozygotes, accumulated lipofuscin in the liver at an early age compared with their wild-type siblings (n = 10 for each group) (Figure 8B–G). Although heterozygous *nrs* ^m/+^ and *psm7* ^m/+^ mutant fish did not display significantly increased SA-β-gal levels at 25 months (2.1 years) and 18 months (1.5 years) of age, respectively (Figure 8A), both of these mutants did show increased lipofuscin accumulation at these ages (*nrs*, n = 9 and *psm7*, n = 8) (Figure 8E, G). This indicated that different aging phenotypes may occur independently or that liver lipofuscin buildup may be an earlier or more sensitive indicator of aging in these mutants. Taken together, these results suggest that our newly identified mutants show a robust early-onset expression of aging biomarkers that normally only manifest in much older wild-type animals.

A striking phenotype associated with the *terf2* ^m/m^ homozygous mutant embryos was observed in the central nervous system, including the eye (retina) and brain, as shown in Figure 4C–E and Figure 7W–Y. Given the degenerative phenotype seen in embryonic *terf2* ^m/m^ retinas (Figure 4C–E, right panels), we examined histological sections of heterozygous adult mutants. Histological sections of mutant (n = 23) and wild-type sibling (n = 15) retinas were analyzed at various ages to determine the cellular basis for the observed age-dependent retinal defects. We observed structural abnormalities, principally retinal cell degeneration, in most aged mutant retinas with drusen-like autofluorescent accumulations around the retinal pigment epithelium (RPE). This degeneration was not uniform over the retinas but tended to be patchy. Areas of rods (rod outer segments) degeneration were interspersed between areas where significant numbers of rods remained. Cones (cone outer segments) generally were better preserved than the rods, but areas of cone degeneration were also noted. The observed degeneration was progressive with age. In animals older than 12 months, degeneration was usually seen only in the central-most regions of the retina. By 21 months of age, however, degeneration was observed across much of the retina, although the far peripheral regions tended to be spared. Importantly, in the wild-type zebrafish retinas, these degenerative changes appeared dramatically with advancing age (Figure S7B).

Representative histological sections from a 23-month old wild-type sibling fish and an age-matched heterozygous *terf2* ^m/+^ mutant fish were compared and are shown in Figure 8I. In zebrafish, the photoreceptors are typically tiered so that in the light-adapted retina, the rods are positioned more distally than the cones. In the 23-month old *terf2* ^m/+^ retina, the peripheral regions were found to be similar to control retinas, but obvious abnormalities were observed throughout the central retina. In some areas only the rods were affected, i.e., the rod outer segments were disorganized and reduced in length, and autofluorescent accruals in the RPE were increased in number and size (Figure 8I, right panel in *terf2* ^m/+^). In other areas, the rods were severely degraded but the cones appeared relatively normal. In yet other areas, both the cones and rods were affected, i.e., both were sometimes disorganized and reduced in length (data not shown). Moreover, the inner plexiform layers of the retina usually looked thinner in affected animals, and occasionally some patchy thinning of the inner nuclear layer and empty spaces were also observed (Figure 8I, left panel in *terf2* ^m/+^), suggesting some loss of inner retinal neurons. To investigate whether there might be loss of elements other than photoreceptors in the mutant zebrafish retinas, we measured the thickness of various retinal layers centrally in control (n = 6) and mutant (n = 8) animals at 23 months of age (Table S2), and also in control (n = 6) and mutant (n = 6) animals at 30 months of age (data not shown). The retinas of mutant animals were clearly thinner than those of the wild-type fish (Figure 8I). Much of this change in thickness was accounted for by a decrease in the thickness of the photoreceptor layer and inner plexiform layer.

**Figure 9:**
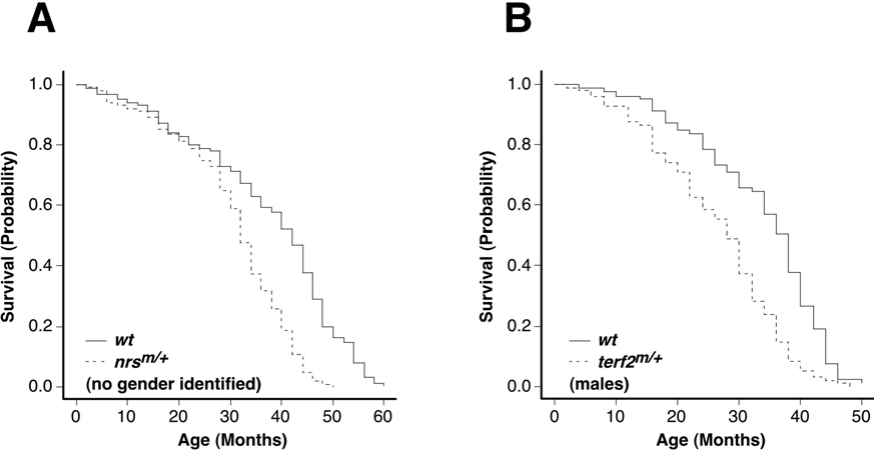
Heterozygous *nrs* and *terf2* mutant zebrafish showing a shorter lifespan in adults. Shorter lifespan in heterozygous *nrs* ^m/+^ and *terf2* ^m/+^ mutants is demonstrated by Kaplan-Meier survival analysis. Survival curves of cohorts of no gender identified heterozygous *nrs* ^m/+^ (n = 256) and their wild-type siblings (n = 148) (A), and male heterozygous *terf2* ^m/+^ (n = 79) and their wild-type male siblings (n = 96) (B), are shown.

Finally, we obtained Kaplan-Meier survival curves for some of our mutant fish. The oldest fish that were heterozygous for *psm* mutations in our stocks at the time of writing had not reached their maximum lifespan (wild-type zebrafish have a maximum lifespan of roughly 5 years [51]), so we have not yet been able to determine the effects of these mutations upon overall lifespan. We have, however, maintained populations of heterozygous *nrs* and *terf2* mutant fish and their wild-type siblings until death, and conducted observational studies on their lifespan. We scored the cohorts of *nrs* ^m/+^ fish (no genders identified; n = 148) in comparison with their wild-type siblings (no genders identified; n = 256), and found significant decreases in the lifespan of the heterozygous mutants (*P*<0.0001, log rank test) (Figure 9A). Moreover, the male *terf2* ^m/+^ fish cohorts (n = 96) also manifested a shorter lifespan compared with that of their wild-type male siblings (n = 79) (*P*<0.0001, log rank test) (Figure 9B). In contrast, neither the heterozygous *terf2* mutant females nor their wild-type female siblings had reached the end point of their lifespan during the period of our current experiment. These lifespan analyses of the two mutations identified by embryonic senescence phenotypes suggest that screening for the embryonic appearance of ‘aging biomarkers’ may in some cases at least, predict a role for specific genes in the organismal aging process.

## Discussion

The goal of our current study was to test the hypothesis that mutations which enhance the appearance of embryonic stress markers might result in degenerative or even aging phenotypes in adults. We have presented the results of two different screens for potential aging mutants in this report, both utilizing SA-β-gal production in the zebrafish embryo as a key part of the screening process. We first screened a collection of retrovirus-mediated insertional mutants for elevated SA-β-gal production in unstressed homozygous embryos. Notably, this collection of insertional mutants was isolated based on the requirement for homozygous developmental phenotypes. Each of the two mutants out of the 11 candidates from this first screen included a lesion in the *nrs* gene, the zebrafish homologue of the *Drosophila* spinster gene a known regulator of aging in flies [33], or the *terf2* gene, which plays roles in telomere protection and telomere-length regulation as a component of the telosome/shelterin complex [35]. In the second screen, using an oxidant agent as a sensitizer, we have isolated a series of mutants, denoted *psm* mutants, which showed elevated SA-β-gal expression in the stressed heterozygous state and additionally in an unstressed homozygous state of embryos. Sensitized screens are much less labor and time intensive than traditional screens for recessive phenotypes in homozygous mutant embryos. Importantly, heterozygous mutant fish from both screens showed elevated expression of aging biomarkers in relatively younger ages. Thus, mutants from both screens showed degenerative phenotypes in homozygous embryos during early development and in heterozygous adults with age. Notably, the heterozygous animals in some cases also appeared to die at earlier ages than controls.

It is possible that the two screens revealed some different classes of mutants. For instance neither of the two insertional mutants which we studied in detail showed statistically significant alterations in their sensitivity to oxidative stress as heterozygotes (data not shown). The heterogeneity of the observed phenotypes in mutants from the 2 screens persisted in the adult heterozygotes. Both *nrs* and *psm* mutations showed hastened lipofuscin accrual in the livers of young adult heterozygotes, whereas two *psm* mutants (*psm6* and *psm9*) showed enhanced SA-β-gal production in relatively young adults (1.5 y) compared with their wild-type siblings, a trait shown only in older *nrs* ^m/+^ fish (3.3 y). However, since we examined mutants for only two genes in each screen in any detail, it is premature to derive substantial conclusions at this stage.

The accelerated production of lipofuscin in the liver is a phenotype that was found to be common to the adult zebrafish from both screens. Lipofuscin accumulation is a hallmark of aging in many organisms from worms to mammals [32],[52]. Tissues that are traditionally thought to be sensitive to lipofuscin accrual in mammals include the brain, and both skeletal and cardiac muscle. However, previous studies from our laboratory have shown that there is no clear accumulation of lipofuscin inclusions with age in wild-type zebrafish skeletal muscle cells and cardiac myocytes, at least up to 2.5 years of age [13],[19] (unpublished observations). In contrast, age-related changes in liver structure and lipofuscin accumulation have been demonstrated in male mice [50]. We have also observed premature lipofuscin accrual in the adult liver of our heterozygous male *psm* mutants as well as in male *nrs* ^m/+^ heterozygotes. It is widely believed that oxidative damage processes underlie sources of lipofuscin production, and that its accumulation may have multiple negative effects, including a further increase in sensitivity to many stress-induced damage responses [32]. Since we observed no obvious response to exogenous (extrinsic) oxidative stress by BHP in *nrs* mutant embryos, we suspect that differences in intrinsic energy metabolism may underlie lipofuscin appearance in the *nrs* mutants, although this clearly requires further investigations. In the case of flies, it has also been shown that mutations in the *spinster* gene cause enhanced lipofuscin production [33], though the precise underlying mechanism also remains unknown. Therefore, parallel studies of this gene product in other organismal aging model systems would be very desirable to provide a more conclusive insight into its mechanistic roles.

There is cumulative evidence to date to suggest that early onset neuronal degeneration phenotypes in homozygous zebrafish mutants are predictive of a late-onset visual impairment in the corresponding heterozygous animals [53]–[56]. Intriguingly, in heterozygous *terf2* ^m/+^ mutant adult fish, drusen-like autofluorescent accumulation (presumably caused by lipofuscin accrual) is more obvious in the RPE compared with age-matched siblings. In contrast to other tissues, ocular lipofuscin has been identified as *N*-retinylidene-*N*-retinylethanolamine (A2E). A2E is a quaternary amine and retinoid by-product of the visual cycle and causes the accumulation of free and esterified cholesterol in RPE cells [57],[58]. Although endogenously produced A2E in the RPE has been associated with macular degeneration, the precise mechanisms are unclear. Therefore, the involvement of the telomeric factor TRF2 in the mechanism of the RPE lipofuscin accrual might provide intriguing new insights into potential novel strategies for the prevention and treatment of neurodegenerative disorders. Alternatively, telomere-associated proteins might be involved in neural differentiation, as homozygous *terf2* ^m/m^ mutant embryos show an aggressive neurodegenerative phenotype in both the eye (retina) and brain. In this regard, divergent molecular and physiological responses to telomere dysfunction in mitotic neural stem/precursor cells and postmitotic neurons appear to regulate the differentiation and survival of neurons as well as RPE cells [47],[48].

Notably, both *terf2* (males) and *nrs* (gender not identified) heterozygous mutant had shorter lifespans in comparison with their control wild-type siblings, suggesting that partial loss-of-function/decrease-of-function in these genes may have systemic effects on physiological aging rather than the organ-specific tissue aging on which we focused in the current study. In addition, there may be gender-dependent differences in longevities and mortality rates in zebrafish when we look at the survival curves of wild-type siblings of *terf2* and *nrs* mutants. Male *terf2* heterozygous mutant fish and their wild-type male siblings were kept isolated from females except for the occasional matings performed in our current study. Heterozygous *terf2* mutant females and their wild-type female siblings had not reached the end point of their lifespan at the time when the male fish all died out. On the other hand, heterozygous *nrs* mutant males and females basically co-habited throughout their life, as did their wild-type siblings. When they died, sex determination was therefore not possible due to rapid decomposition (autolysis) in water. Although further studies are definitely needed, social and environmental factors between genders might affect zebrafish lifespan in addition to their genetic background.

Any understanding of the mechanisms by which the *psm* genes regulates the functions of age accompanied with degenerative phenotypes will require positional cloning of the mutated genes. The *psm* mutants may also be useful for studying signal transduction pathways and related biological processes in addition to both stress responses and aging. The fact that these mutations regulate embryonic tissue-specific responses to oxidative stress is also interesting per se. It will be of considerable interest to see if these mutations lie in genes already associated with oxidant responses, such as antioxidant genes or genes that maintain mitochondrial functions. It is also possible that the *psm* mutants which showed neuronal phenotypes may have value as new models for specific disorders such as Huntington's disease, Parkinson's disease, Alzheimer's disease, amyotrophic lateral sclerosis, and ataxia telangiectasia, because several lines of evidence suggest that oxidative stress is associated with the development of these neurodegenerative diseases [59]–[63]. In addition, oxidative stress has been shown to play a role in sarcopenia and other muscle wasting conditions [5],[6], where the *psm7* mutant may be involved. Since we have already shown an embryonic haplosensitivity of the *psm* mutants to oxidative stress by the very nature of the CASH strategy, we are currently further investigating the possibility that more of these mutants may show accelerated onset of aging in the adult heterozygotes, expanding their utility as models of stress-associated pathophysiological aging and degenerative disease.

In the future, we hope to further utilize our technology to identify a real “suppressor” type mutant which would display enhanced embryonic stress resistance and, perhaps, a longer healthy lifespan (‘health span’). Alternatively, it might also be possible to isolate a ‘revertant’ from the background of an accelerated aging mutant, which would restore the original normal phenotype by means of a suppressor mutation.

In summary, our current study has demonstrated for the first time in vertebrates that it is possible to obtain mutations that alter adult aging markers/phenotypes and lifespans by screening mutagenized and sensitized embryos for the extemporal expression of the aging biomarker. It is our hope that this novel tactic of screening for aging biomarkers in zebrafish embryos may open a new avenue for the future genetic dissection of vertebrate aging mechanisms.

## Materials and Methods

### Zebrafish Husbandry, Maintenance, and Longevity

Zebrafish of the wild-type strain and mutants were maintained with a 14:10 h light/dark cycle and fed living brine shrimp twice per day. Brine shrimp was given using 1 mL pipettes at an amount of about 0.75 mL per 20 fish. Flake food was also given a few days per week semiquantitatively according to the number of fish in the tanks. A continuously cycling Aquatic Habitats™ system was used to maintain water quality (Apopka, FL, USA) which completely replaces the water in each tank every 6–10 min. Each tank is a baffle/tank system that ebbs water in a circular motion to ensure flushing and water turnover. Ultraviolet (UV) sterilizers (110,000 microwatt-s cm^−2^) were employed to disinfect the water and prevent the spread of disease in the recirculating system. The water temperature was maintained at 28±0.5°C. The system continuously circulated water from the tanks through Siporax™ strainers, through a fiber mechanical filtration system, and finally into a chamber containing foam filters and activated carbon inserts. Water quality was tested daily for chlorine, ammonia, pH, nitrate, and conductivity under real-time computer monitoring with alarms to signal potential fluctuations. The general health of each fish was observed on a daily basis throughout fish life to monitor longevity, and abnormal looking or acting fish were quarantined into isolated tanks unconnected from the general circulation. The water in these quarantined tanks was treated with methylene blue (0.0001–0.001%). If and when fish recovered, they were returned to their original tanks on the general circulation. All animals showing signs of infectious or parasitic disease that are not alleviated by a 7-day incubation with methylene blue in water were euthanized in a beaker containing tricaine (approximately final 0.5% in water). Animals exhibiting overt tumors or extreme morbidity were also euthanized.

Embryos were collected by natural spawning, raised in 10% Hank's saline with or without 0.003% 1-phenyl-2-thiourea (PTU) (embryo media) and staged according to Kimmel et al. [64]. All embryos were incubated at 28.5°C during development.

### Quantitative Analysis and Statistics

Data processing and statistical analyses were performed using Microsoft Excel and Statistical Package for the Social Sciences (SPSS) version 14.0, which were used to generate each of the scatter plots, tables, and graphs shown in the text, performing statistical tests where appropriate. Additional statistical analyses were performed at the Department of Biostatistics and Computational Biology, Dana-Farber Cancer Institute. These analyses included survival estimates using the method of Kaplan and Meier, and comparison of survival between mutant fish and their wild type controls using the log rank test.

### SA-β-gal Assay and Quantitation

Zebrafish adults and embryos were fixed in 4% paraformaldehyde in phosphate buffered saline (PBS) at 4°C (for 3 days in adults and overnight in embryos), and then washed 3 times for 1 h in PBS-pH 7.4 and for a further 1 h in PBS-pH 6.0 at 4°C. Staining was performed overnight at 37°C in 5 mM potassium ferrocyanide, 5 mM potassium ferricyanide, 2 mM MgCl_2_, and 1 mg/ml X-gal in PBS adjusted to pH 6.0. All animals were photographed under the same conditions using reflected light under a dissecting microscope. SA-β-gal activity in each animal was quantitated using a selection tool in Adobe Photoshop for a color range that was chosen by 25 additive blue color selections of regions that showed visually positive SA-β-gal staining. For analyses of embryos, these regions were selected in each embryo proper only and not in the yolk in order to eliminate variability due to differences in initial yolk volume and yolk consumption over time. Since the yolk stains much more intense blue for SA-β-gal at all stages of development than any other embryonic tissues, even under conditions of high oxidative stress, it was desirable to eliminate this as a source of variability. Following pixel selection, a fuzziness setting of 14 was used, and the chosen pixel number was calculated using the image histogram calculation. For adult zebrafish analyses, the trunk area for colorimetric quantitation was chosen by selection of the area between the operculum and the dorsal and anal fins (Figure S1).

### Molecular Analysis and Gene Knockdown

The cloning of zebrafish catalase cDNA was performed using the SuperScript One-Step RT-PCR kit with Platinum Taq (Invitrogen) according to the manufacturer's instructions, by using the forward primer 5′-TTTGCCTCGTGTTTTGTCAC-3′ and reverse primer; 5′-GGAGTCAGTGTTGCATTTGCT-3′. These primers were designed using the flanking regions of the zebrafish Ensembl sequence for the predicted human catalase homolog. The full-length cDNA was then cloned into the pCS2+ vector. The resulting plasmid was linearized by digestion at a restriction site immediately after the poly-A signal, and capped mRNAs were transcribed *in vitro* using the mMachine Kit (Ambion Inc.) following the manufacturer's instructions. The stated amount (300 pg) of mRNA were injected into one-cell stage zebrafish embryos using a gas driven microinjector (Medical Systems Corp.).

Knockdown of zebrafish *catalase* was performed by injection of 8 ng antisense morpholino oligonucleotide (MO) (Gene-Tools, LLC) with the sequence 5′-TCGACTTTTCTCTGTCGTCTGCCAT-3′ or a control morpholino 5′-CCTCTTACCTCAGTTACAATTTATA-3′. Knockdown of zebrafish *nrs/spns1* and *terf2/terfa* was performed also by injections of MOs (8 ng) containing the sequences 5′-ATCTGCTTGTGACATCACTGCTGGA-3′ and 5′-GGTTCGCAGGGTTTGTCGCTCATTC-3′, respectively.

### Screening of Retrovirus-Mediated Insertional Mutants

Heterozygous incrosses of each mutant line were performed, and the resulting embryos were raised in 10% Hank's saline at 28.5°C. The embryos were fixed at either 3.5 dpf or at least 18 h before the occurrence of embryonic lethality after 3.5 dpf for lines whose the homozygotes are known to die. SA-β-gal staining was performed as above, and positive candidates were determined by correlating high SA-β-gal activity with the presence of a homozygous mutant phenotype. Since some of mutants needed to be distinguished by their pigmentation patterns, the entire screening of 306 mutants was performed in these embryos without PTU treatment. Embryos of selected 11 candidates were also processed for single-embryo SA-β-gal quantitation with PTU treatment as described above.

### ENU Mutagenesis

ENU mutagenesis was performed as previously described [65]. Briefly, regularly bred 1-year old *AB strain adult zebrafish males were treated with 3 mM N-ethyl-N-nitrosourea at 20°C for 1 h once a week for 3 times. From five to seven weeks after the final treatment, mutagenized males were outcrossed to wild-type *AB females and F_1_ progeny were raised to adulthood.

### Peroxide CASH Screening

F_1_ mutagenized males were outcrossed with three wild-type *AB females to maximize the resulting clutch size. 50 embryos from the resultant clutches were then raised from 6 hpf to 6 dpf in 10% Hank's Saline with 0.003% 1-phenyl-2-thiourea (PTU) and 350 µM tert-butyl hydroperoxide (BHP). The media was refreshed every 48 h. Embryos were processed for single-embryo SA-β-gal quantitation as described above. Clutches showing either obvious phenotypic abnormalities in 50% of the embryos, or having staining standard deviations of at least 1.5 times that of wild-type clutches in both of two independent breedings, were considered to be a positive hit. Positive hit F_1_ males were subsequently outcrossed with wild-type *AB females and the resulting F_2_ generation was raised to adulthood. F_3_ embryos derived from these F_2_ sibling incrosses in each hit family were assessed for their phenotype and SA-β-gal activity levels both in the presence and absence of oxidative stress.

### *In Vivo* Detection of Cell Death, ROS Generation, and Neurodegeneration

For the *in vivo* detection of cell death, live 2-day old embryos were incubated in 2 µg/ml acridine orange (AO) (Sigma) in embryo media in the dark for 30 min and washed three times for 5 min in fresh embryo media. Fluorescence was then observed under a 488 nm wavelength excitation. For *in vivo* ROS detection, live 2–4-day old embryos (2–4 dpf) were incubated in 5 µM 2′,7′-dichlorofluorescein diacetate (DCFH-DA) (Sigma) for 20 min at 28.5°C and washed three times for 5 min with embryo media. Fluorescence was again observed under a 488 nm wavelength excitation. For Fluoro-Jade B histochemical analysis of 2 dpf embryos, adjacent sections were stained using the standard Fluoro-Jade staining procedure as described previously [66].

### Histological Analysis

Embryos and adult tissue samples were fixed in 4% paraformaldehyde in PBS for 48 h at 4°C. Samples were dehydrated in ethanol and infiltrated in JB-4 resin following the manufacturer's instructions (Polysciences Inc.). Specimens were then sectioned at 5 µm using a Jung Supercut 2065 microtome. Histological hematoxylin-eosin (H&E) staining of the sections was subsequently carried out using standard protocols. After digestion with diastase, periodic acid Schiff's (PAS) staining was performed as follows: sections were heat-adhered to slides at 70°C for 5 min and placed in the following solutions at room temperature; 1% periodic acid for 5 min, several water changes over 5 min, Schiff's reagent for 30 min, 0.5% sodium metabisulfite in 1% concentrated HCl 3×2 min, and several water changes for 10 min each.

## Supporting Information

Figure S1 Pixel images for quantitation of SA-β-gal activity in zebrafish. (A–D) Colorimetric quantitation of SA-β-gal activity staining in the trunk sections of adult zebrafish. Lateral photographs were taken, and the area between the operculum and the dorsal and anal fins was chosen for quantitation (A and B). The blue pixel area was calculated (C and D as described in Materials and Methods), and SA-β-gal activity is expressed as a percentage of the total area values. The analysis was performed on both sides of each fish. Shown here are fish aged 5 months (A and C) and 57 months (B and D). E and F: Colorimetric quantitation of SA-β-gal activity in zebrafish embryos. The total blue pixel number was determined from lateral photographs of individual 3.5-day old zebrafish embryos. SA-β-gal staining intensities were quantified in untreated embryos (E) and embryos incubated in 500 mM BHP (F).
(0.40 MB TIF)

Figure S2 Morpholino-induced knockdown of *nrs* and *terf2* in zebrafish embryos generates phenocopies of the corresponding mutants. (A) *nrs* morphants show yolk-opaque phenotypes starting at 2.5 dpf (with PTU), which is an exact phenocopy of the *nrs* mutant embryos which also manifested an obvious yolk-opaque substance. Upon SA-β-gal staining at 3 dpf, an extremely high level of induction was detected in the *nrs* morphants, in an identical manner to the SA-β-gal levels observed in the *nrs* mutants, while the control-injected embryos did not show any significant SA-β-gal activity as well as opaque yolks. (B) *terf2* morphants (3.5 dpf) show robust SA-β-gal induction having relatively moderate but quite a similar morphological phenotype to the *terf2* allele mutant, *terfa* ^hi1182/hi1182^ which has a slightly weaker phenotype than *terfa* ^hi3678/hi3678^.
(0.56 MB TIF)

Figure S3 Retrovirus-insertional mutants showing high SA-β-gal activity. The eleven homozygous 3.5 dpf retrovirus-insertional mutants stained with high SA-β-gal are shown in comparison with a wild-type control (with PTU). All of the information we obtained regarding these mutants is summarized in Table 1.
(0.42 MB TIF)

Figure S4 Retrovirus-insertional mutants showing low SA-β-gal activity. There were several low SA-β-gal intensity insertional mutants such as *hi3820B* (*60S ribosomal protein L11 gene*) (n = 12; 5 dpf), *hi2230* (*eukaryotic translation initiation factor 3, subunit 7 gene*) (n = 15; 4 dpf), and *hi601* (*small nuclear ribonucleoprotein D1 gene*) (n = 14; 3.5 dpf). Representative SA-β-gal stained homozygous embryos (two individuals from each of these three mutant groups) are shown.
(0.20 MB TIF)

Figure S5 Homozygous *psm* mutant embryos showing high levels of tissue-specific SA-β-gal activity without oxidative stress. Homozygous 3.5 dpf neurodegenerative zebrafish *psm* mutants (*psm2, 5, 6, 8, 9, 10, 11*) show much higher levels of punctate SA-β-gal staining throughout their central nervous system compared with wild-type embryos (with PTU). Of note, the muscle atrophy mutant *psm7* shows particularly high levels of punctate staining in the trunk region.
(0.26 MB TIF)

Figure S6 Dystrophin expression is normal in *psm7* ^m/m^ embryos. Immunostaining of dystrophin (red) in muscle myotomes of 3.5 dpf *psm7* ^m/m^ zebrafish embryos (B) is similar to that in wild-type embryos (A), indicating that dystrophin-related muscle attachment is not defective in this mutant. Staining was performed as described by Bassett et al. [67] with the exception that the secondary antibody used was Alexa Fluor 594-conjugated anti-mouse IgG at a 1∶1000 dilution.
(0.08 MB JPG)

Figure S7 Liver and eye histology in the adult zebrafish with age. (A) Liver sections of 1 y, 2.1 y, 2.8 y, and 3.8 y wild-type zebrafish were stained by H&E or PAS staining. Hepatocyte density and eosinophilic staining can be seen to increase with age by H&E staining. PAS-positive staining for lipofuscin also shows increased levels of this biomarker in aging liver tissue. (B) Eye sections of 5-month, 20-month, 36-month, and 58-month old wild-type fish were stained by H&E. In the light adapted retina, the rods (r) sit distally to the cones (c); ‘in’ indicates the inner nuclear layer, and ‘ip’ indicates the inner plexiform layer. Processes from the pigment epithelium (PE) extend between the outer segments of the rods. Aged (58-month old) wild-type fish show increased drusen-like accruals (yellow arrows) with autofluorescence in the RPE (lower right panel), compared with younger (20-month old) wild-type fish (upper right panel). (Scale bar: 100 µm.)
(1.30 MB JPG)

Table S1 Complementation tests between *psm*, *nrs*, and *terf2* mutants.
(0.05 MB DOC)

Table S2 Retinal thickness in heterozygous *terf2* mutants and their wild-type siblings.
(0.04 MB DOC)
