# Supplementary figures and images for: The Identification of Zebrafish Mutants Showing Alterations in Senescence-Associated Biomarkers
Source: PLoS Genet. 2008 Aug 15;4(8):e1000152. doi: 10.1371/journal.pgen.1000152 (PMC2515337; doi:10.1371/journal.pgen.1000152)

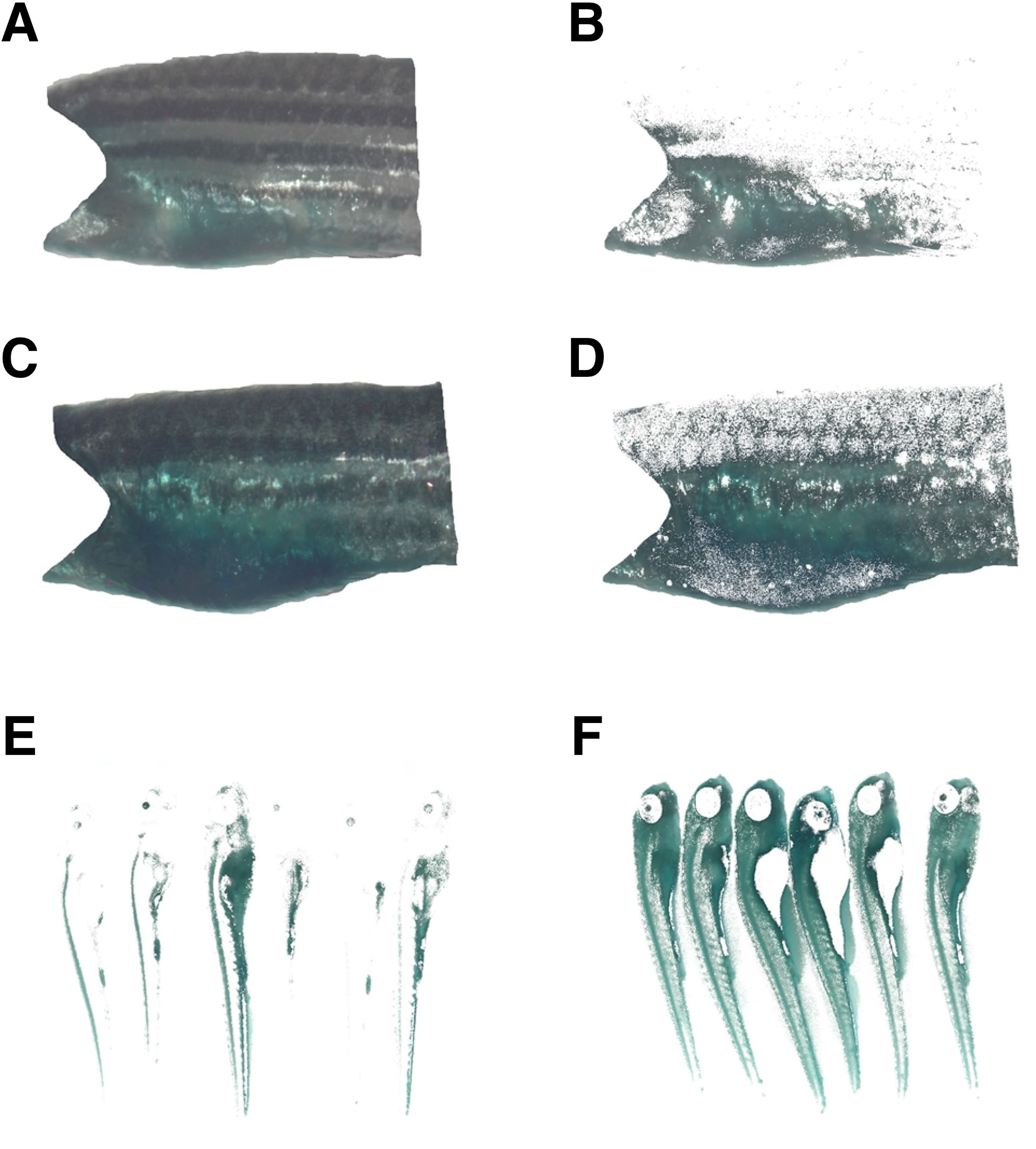

Supplement: Figure S1 — Pixel images for quantitation of SA-β-gal activity in zebrafish. (A–D) Colorimetric quantitation of SA-β-gal activity staining in the trunk sections of adult zebrafish. Lateral photographs were taken, and the area between the operculum and the dorsal and anal fins was chosen for quantitation (A and B). The blue pixel area was calculated (C and D as described in Materials and Methods), and SA-β-gal activity is expressed as a percentage of the total area values. The analysis was performed on both sides of each fish. Shown here are fish aged 5 months (A and C) and 57 months (B and D). E and F: Colorimetric quantitation of SA-β-gal activity in zebrafish embryos. The total blue pixel number was determined from lateral photographs of individual 3.5-day old zebrafish embryos. SA-β-gal staining intensities were quantified in untreated embryos (E) and embryos incubated in 500 mM BHP (F). (0.40 MB TIF) [file pgen.1000152.s001.tif]

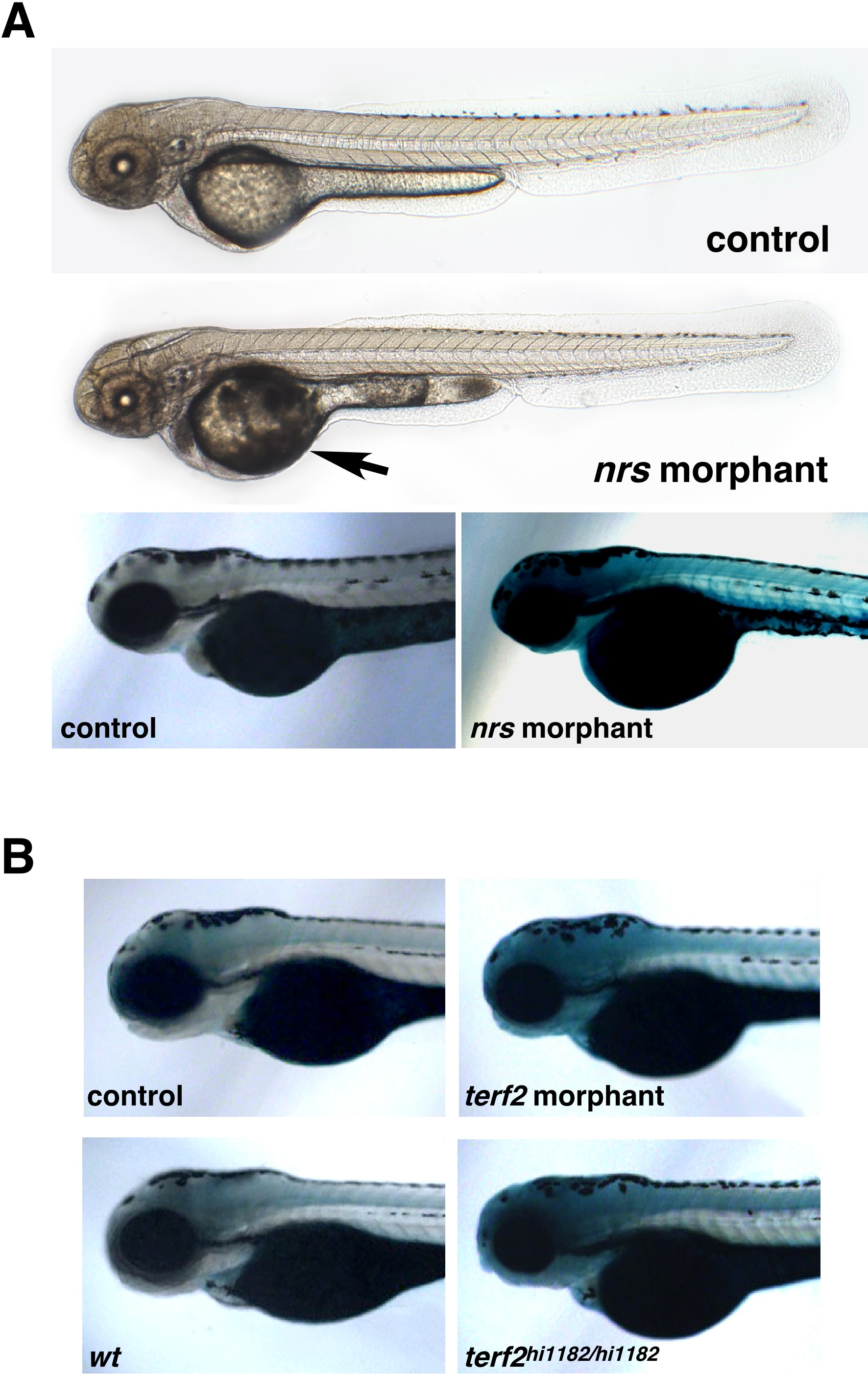

Supplement: Figure S2 — Morpholino-induced knockdown of nrs and terf2 in zebrafish embryos generates phenocopies of the corresponding mutants. (A) nrs morphants show yolk-opaque phenotypes starting at 2.5 dpf (with PTU), which is an exact phenocopy of the nrs mutant embryos which also manifested an obvious yolk-opaque substance. Upon SA-β-gal staining at 3 dpf, an extremely high level of induction was detected in the nrs morphants, in an identical manner to the SA-β-gal levels observed in the nrs mutants, while the control-injected embryos did not show any significant SA-β-gal activity as well as opaque yolks. (B) terf2 morphants (3.5 dpf) show robust SA-β-gal induction having relatively moderate but quite a similar morphological phenotype to the terf2 allele mutant, terfa hi1182/hi1182 which has a slightly weaker phenotype than terfa hi3678/hi3678. (0.56 MB TIF) [file pgen.1000152.s002.tif]

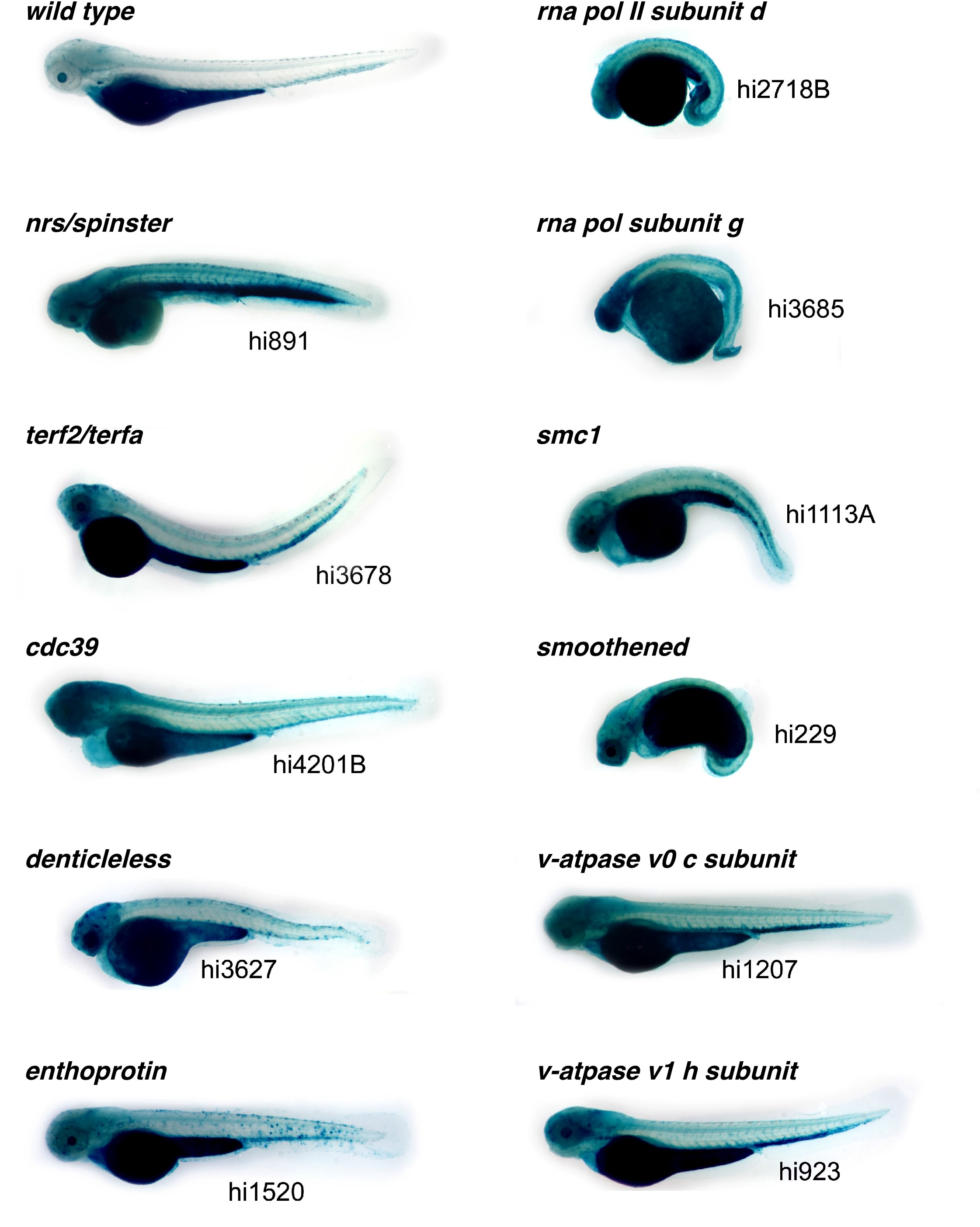

Supplement: Figure S3 — Retrovirus-insertional mutants showing high SA-β-gal activity. The eleven homozygous 3.5 dpf retrovirus-insertional mutants stained with high SA-β-gal are shown in comparison with a wild-type control (with PTU). All of the information we obtained regarding these mutants is summarized in Table 1. (0.42 MB TIF) [file pgen.1000152.s003.tif]

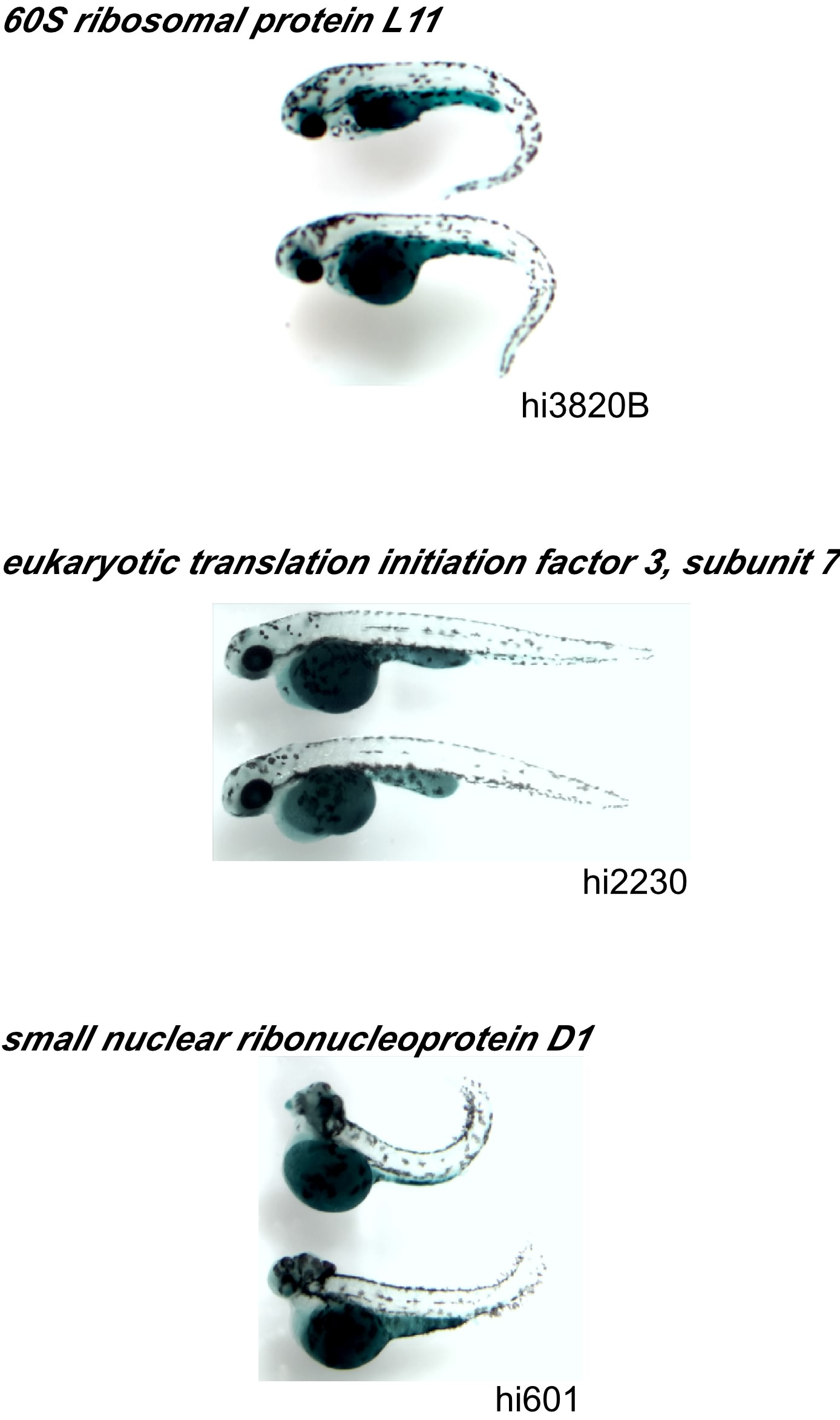

Supplement: Figure S4 — Retrovirus-insertional mutants showing low SA-β-gal activity. There were several low SA-β-gal intensity insertional mutants such as hi3820B (60S ribosomal protein L11 gene) (n = 12; 5 dpf), hi2230 (eukaryotic translation initiation factor 3, subunit 7 gene) (n = 15; 4 dpf), and hi601 (small nuclear ribonucleoprotein D1 gene) (n = 14; 3.5 dpf). Representative SA-β-gal stained homozygous embryos (two individuals from each of these three mutant groups) are shown. (0.20 MB TIF) [file pgen.1000152.s004.tif]

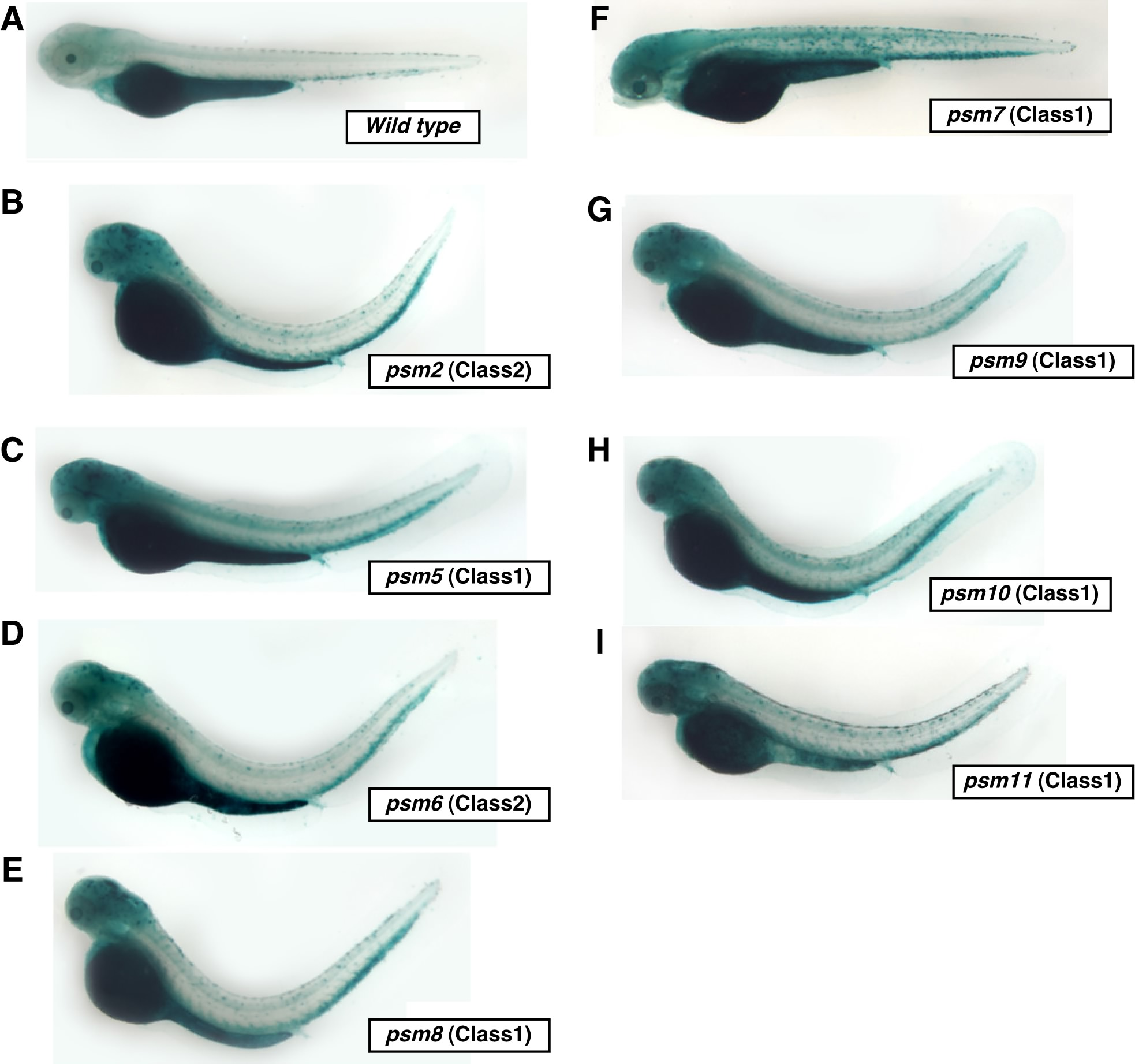

Supplement: Figure S5 — Homozygous psm mutant embryos showing high levels of tissue-specific SA-β-gal activity without oxidative stress. Homozygous 3.5 dpf neurodegenerative zebrafish psm mutants (psm2, 5, 6, 8, 9, 10, 11) show much higher levels of punctate SA-β-gal staining throughout their central nervous system compared with wild-type embryos (with PTU). Of note, the muscle atrophy mutant psm7 shows particularly high levels of punctate staining in the trunk region. (0.26 MB TIF) [file pgen.1000152.s005.tif]

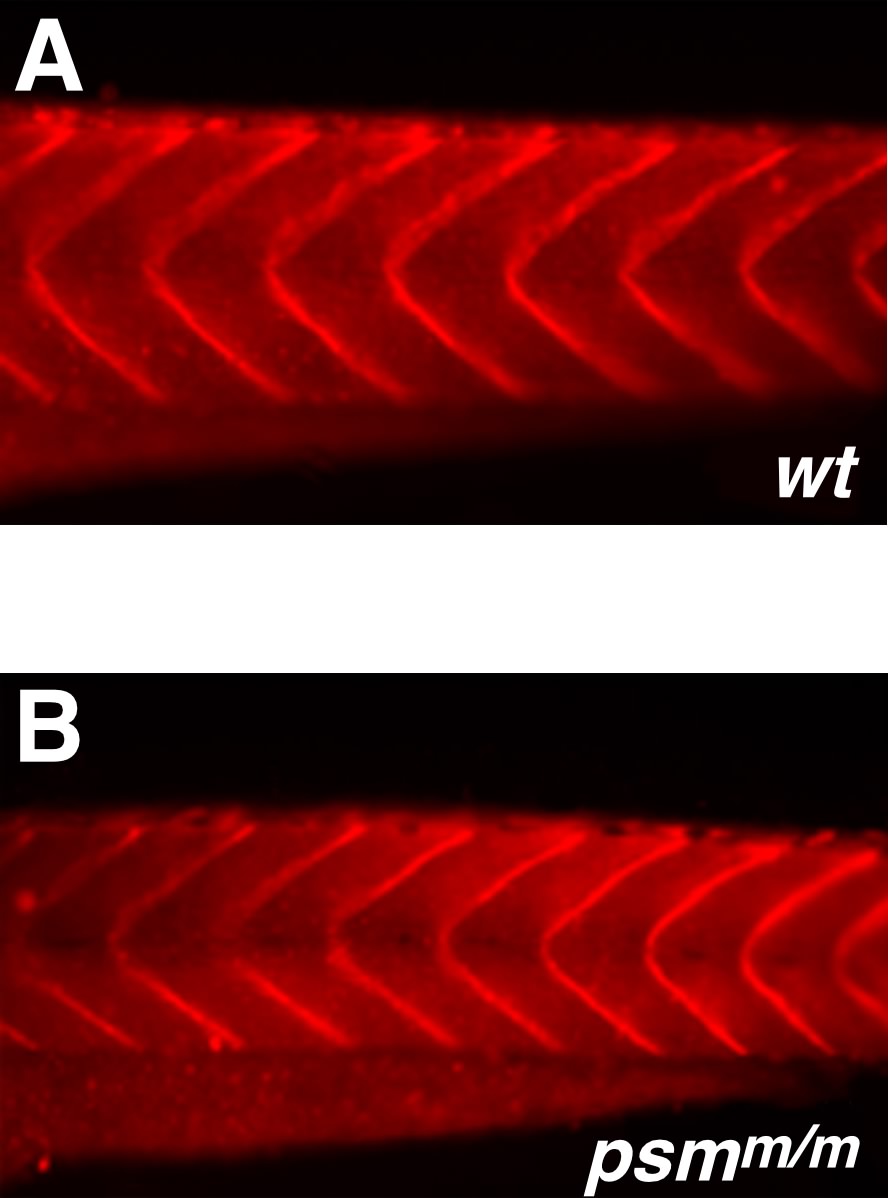

Supplement: Figure S6 — Dystrophin expression is normal in psm7 m/m embryos. Immunostaining of dystrophin (red) in muscle myotomes of 3.5 dpf psm7 m/m zebrafish embryos (B) is similar to that in wild-type embryos (A), indicating that dystrophin-related muscle attachment is not defective in this mutant. Staining was performed as described by Bassett et al. [67] with the exception that the secondary antibody used was Alexa Fluor 594-conjugated anti-mouse IgG at a 1∶1000 dilution. (0.08 MB JPG) [file pgen.1000152.s006.jpg]

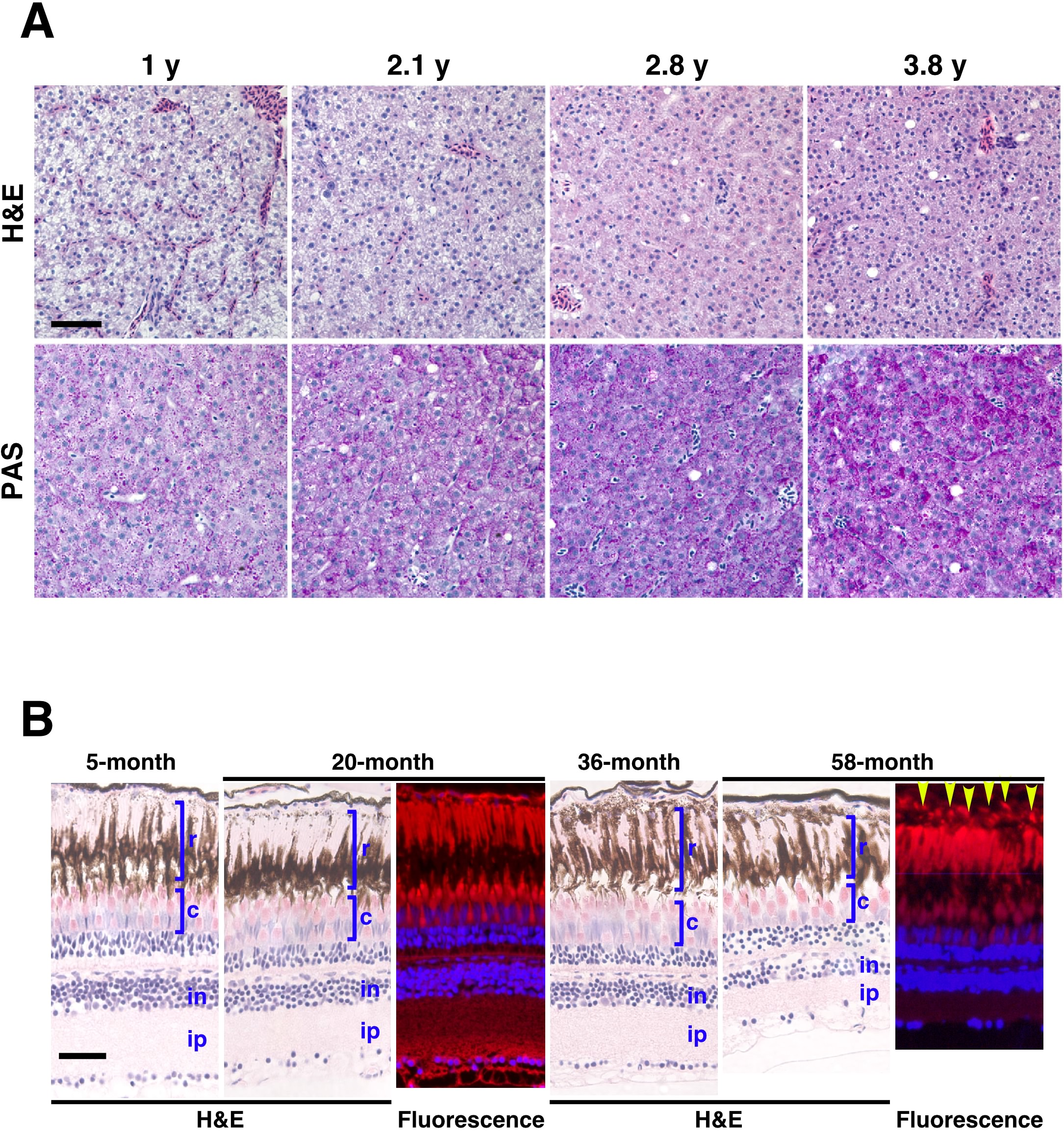

Supplement: Figure S7 — Liver and eye histology in the adult zebrafish with age. (A) Liver sections of 1 y, 2.1 y, 2.8 y, and 3.8 y wild-type zebrafish were stained by H&E or PAS staining. Hepatocyte density and eosinophilic staining can be seen to increase with age by H&E staining. PAS-positive staining for lipofuscin also shows increased levels of this biomarker in aging liver tissue. (B) Eye sections of 5-month, 20-month, 36-month, and 58-month old wild-type fish were stained by H&E. In the light adapted retina, the rods (r) sit distally to the cones (c); ‘in’ indicates the inner nuclear layer, and ‘ip’ indicates the inner plexiform layer. Processes from the pigment epithelium (PE) extend between the outer segments of the rods. Aged (58-month old) wild-type fish show increased drusen-like accruals (yellow arrows) with autofluorescence in the RPE (lower right panel), compared with younger (20-month old) wild-type fish (upper right panel). (Scale bar: 100 µm.) (1.30 MB JPG) [file pgen.1000152.s007.jpg]
