# Supplementary material for: The Identification of Zebrafish Mutants Showing Alterations in Senescence-Associated Biomarkers
Source: PLoS Genet. 2008 Aug 15;4(8):e1000152. doi: 10.1371/journal.pgen.1000152 (PMC2515337; doi:10.1371/journal.pgen.1000152)
Supplement: Table S1 — Complementation tests between psm, nrs, and terf2 mutants. (0.05 MB DOC) [file pgen.1000152.s008.doc]

**Table S1. Complementation tests between *psm*, *nrs*, and *terf2* mutants.**

| |  | *psm2* | *psm5* | *psm6* | *psm7* | *psm8* | *psm9* | *psm10* | *psm11* | *nrs* | *terf2* | | --- | --- | --- | --- | --- | --- | --- | --- | --- | --- | --- | | *psm2* | N/A | - | - | + | - | - | - | - | + | + | | *psm5* |  | N/A | - | + | - | - | - | - | + | + | | *psm6* |  |  | N/A | + | - | - | - | - | + | + | | *psm7* |  |  |  | N/A | + | + | + | + | + | + | | *psm8* |  |  |  |  | N/A | - | - | - | + | + | | *psm9* |  |  |  |  |  | N/A | - | - | + | + | | *psm10* |  |  |  |  |  |  | N/A | - | + | + | | *psm11* |  |  |  |  |  |  |  | N/A | + | + | | *nrs* |  |  |  |  |  |  |  |  | N/A | + | | *terf2* |  |  |  |  |  |  |  |  |  | N/A |   -, no complementation; +, complementation; N/A, not applicable, n > 50 in each examination with three times repeated independently. |
| --- | --- | --- | --- | --- | --- | --- | --- | --- | --- | --- | --- | --- | --- | --- | --- | --- | --- | --- | --- | --- | --- | --- | --- | --- | --- | --- | --- | --- | --- | --- | --- | --- | --- | --- | --- | --- | --- | --- | --- | --- | --- | --- | --- | --- | --- | --- | --- | --- | --- | --- | --- | --- | --- | --- | --- | --- | --- | --- | --- | --- | --- | --- | --- | --- | --- | --- | --- | --- | --- | --- | --- | --- | --- | --- | --- | --- | --- | --- | --- | --- | --- | --- | --- | --- | --- | --- | --- | --- | --- | --- | --- | --- | --- | --- | --- | --- | --- | --- | --- | --- | --- | --- | --- | --- | --- | --- | --- | --- | --- | --- | --- | --- | --- | --- | --- | --- | --- | --- | --- | --- | --- |
