# Supplementary material for: The Identification of Zebrafish Mutants Showing Alterations in Senescence-Associated Biomarkers
Source: PLoS Genet. 2008 Aug 15;4(8):e1000152. doi: 10.1371/journal.pgen.1000152 (PMC2515337; doi:10.1371/journal.pgen.1000152)
Supplement: Table S2 — Retinal thickness in heterozygous terf2 mutants and their wild-type siblings. (0.04 MB DOC) [file pgen.1000152.s009.doc]

**Table S2. Retinal thickness in heterozygous *terf2* mutants and their wild-type siblings.**

| |  | **Wild type**  (µm) | **TRF2^m/+^**  (µm) | *P*-value | | --- | --- | --- | --- | | Total | 651.17 ± 27.64 | 563.62 ± 27.42 | < 0.0001 | | PL | 319.83 ± 16.22 | 291.75 ± 11.00 | < 0.005 | | ONL | 31.67 ± 5.13 | 29.00 ± 4.21 | = 0.3 | | OPL | 45.17 ± 6.40 | 36.63 ± 3.81 | < 0.01 | | INL | 74.67 ± 7.17 | 62.13 ±4.97 | < 0.005 | | IPL | 141.83 ± 28.31 | 94.13 ± 15.38 | < 0.005 |   Data shown as the mean ± SD. Total, total retina; PL, photoreceptor layer; ONL, outer nuclear layer; OPL, outer plexiform layer; INL, inner nuclear layer; IPL, inner plexiform layer |
| --- | --- | --- | --- | --- | --- | --- | --- | --- | --- | --- | --- | --- | --- | --- | --- | --- | --- | --- | --- | --- | --- | --- | --- | --- | --- | --- | --- | --- |
